# Abscisic acid-responsive transcription factors PavDof2/6/15 mediate fruit softening in sweet cherry

**DOI:** 10.1093/plphys/kiac440

**Published:** 2022-09-21

**Authors:** Zefeng Zhai, Yuqin Xiao, Yanyan Wang, Yueting Sun, Xiang Peng, Chen Feng, Xiang Zhang, Bingyang Du, Xin Zhou, Chao Wang, Yang Liu, Tianhong Li

**Affiliations:** Department of Pomology, College of Horticulture, China Agricultural University, Beijing 100193, China; Department of Pomology, College of Horticulture, China Agricultural University, Beijing 100193, China; Department of Pomology, College of Horticulture, China Agricultural University, Beijing 100193, China; Department of Pomology, College of Horticulture, China Agricultural University, Beijing 100193, China; Department of Pomology, College of Horticulture, China Agricultural University, Beijing 100193, China; Department of Pomology, College of Horticulture, China Agricultural University, Beijing 100193, China; Department of Pomology, College of Horticulture, China Agricultural University, Beijing 100193, China; Department of Pomology, College of Horticulture, China Agricultural University, Beijing 100193, China; Department of Pomology, College of Horticulture, China Agricultural University, Beijing 100193, China; Department of Pomology, College of Horticulture, China Agricultural University, Beijing 100193, China; Department of Pomology, College of Horticulture, China Agricultural University, Beijing 100193, China; Department of Pomology, College of Horticulture, China Agricultural University, Beijing 100193, China

## Abstract

Softening is a key step during fruit ripening that is modulated by the interplay between multiple phytohormones. The antagonistic action of abscisic acid (ABA) and auxin determines the rate of fruit ripening and softening. However, the transcription factors that integrate ABA and auxin signals to regulate fruit softening remain to be determined. In this study, we identified several DNA-binding with One Finger (Dof) transcription factors essential for ABA-promoted fruit softening, based on transcriptome analysis of two sweet cherry (*Prunus avium* L.) varieties with different fruit firmness. We show that PavDof6 directly binds to the promoters of genes encoding cell wall-modifying enzymes to activate their transcription, while PavDof2/15 directly repress their transcription. Transient overexpression of *PavDof6* and *PavDof2/15* in sweet cherry fruits resulted in precocious and delayed softening, respectively. In addition, we show that the auxin response factor PavARF8, the expression of whose encoding gene is repressed by ABA, activates *PavDof2/15* transcription. Furthermore, PavDof2/6/15 and PavARF8 directly bind to the *9-cis-epoxycarotenoid dioxygenase 1* (*PavNCED1*) promoter and regulate its expression, forming a feedback mechanism for ABA-mediated fruit softening. These findings unveil the physiological framework of fruit softening and establish a direct functional link between the ABA–PavARF8–PavDofs module and cell-wall-modifying genes in mediating fruit softening.

## Teaser

Transcriptome analysis identified a set of transcription factors essential for abscisic acid-promoted fruit softening in sweet cherry.

## Introduction

As an economically popular horticultural crop worldwide, sweet cherry (*Prunus avium* L.) is characterized by its attractive appearance when flowering, the delicious taste of its fruits, and the nutrients they contain (Jin et al., 2016). However, the scale of the cherry industry is restricted, in part because the fruits flesh is susceptible to softening and rotting after harvest, and does not withstand storage or transportation, strongly limiting the circulation of this fruit to the consumer market (Wani et al., 2014). Hence, dissecting the mechanisms behind softening is a prerequisite before attempting to modulate this process in sweet cherry.

Softening is one of the hallmarks of ripening in most fleshy fruits. Cell wall modifications are the predominant causes of fruit softening (Fischer and Bennett, 1991; Brummell, 2006). During fruit ripening and softening, cell wall polysaccharides, including cellulose, hemicelluloses, and pectin, are hydrolyzed, leading to the loss of cell wall structures and the reduction of intercellular adhesion (Santiago-Domenech et al., 2008). These processes are modulated by a set of cell wall-modifying enzymes, the most studied of which includes xyloglucan endotransglucosylase/hydrolases (XTHs), polygalacturonases (PGs), pectin methylesterases (PMEs), and pectate lyases (PLs). Pectinases such as PG, PME, and PL catalyze different forms of pectin, leading to changes in pectin solubilization. XTHs promote the depolymerization of hemicellulose. The expression of the genes encoding these cell wall-related enzymes is strongly associated with fruit ripening and softening (Posé et al., 2015; Zhang et al., 2017; Khan et al., 2019). Modifying the expression of these genes influences the fruit firmness and post-harvest shelf-life in tomato (*Solanum lycopersicum*) and strawberry (*Fragaria* × *ananassa*; Quesada et al., 2009; Uluisik et al., 2016; Witasari et al., 2019; Xue et al., 2020). Although the link between cell wall modification enzymes and fruit firmness has been extensively demonstrated in fleshy fruits, the general regulators that determine the expression of their encoding genes and, thus, the initiation of softening have not well been described.

Transcription factors (TFs) have been reported to play roles in fruit softening by regulating the expression of cell wall-related genes. Among them, tomato NON-RIPENING (NOR)-LIKE1 positively regulates fruit softening by activating the expression of *SlPG2a*, *SlPL*, *ENDO-1,4-β-CELLULASE2* (*SlCEL2*), and *EXPANSIN1* (*SlEXP1*; Gao et al., 2018). In banana (*Musa acuminata*), the homeodomain leucine zipper members MaHDZI.4/7/19/26 promote fruit softening by stimulating the transcription of *MaEXP2*/*10*, *MaPG4*, and *MaPL4* (Yang et al., 2020). Additionally, transcriptional repressors downregulate these cell wall modification genes and result in delayed softening. In papaya (*Carica papaya*), ETHYLENE RESPONSE FACTOR9 (CpERF9) inhibits fruit softening by repressing the expression of *CpPME1/2* and *CpPG5* (Fu et al., 2016). In banana, BRASSINAZOLE-RESISTANT1 (MaBZR1) and MaBZR2 delay fruit softening by directly repressing *MaEXP2*, *MaPL2*, and *xyloglucan endotransglucosylase5* (*MaXET5*; Shan et al., 2020). However, despite great strides made in this area of research, additional players likely exist and remain to be identified.

DNA-binding with One Finger (Dof) proteins are among the many well-characterized plant-specific TFs. Members of this family are widely distributed in the plant kingdom and have multiple roles in plant growth and development (Noguero et al., 2013; Gupta et al., 2015). A Dof protein was recently reported to play roles in fruit ripening and softening. Indeed, knockdown of *SlDof1* delayed ripening-related processes, indicating a positive role for SlDof1 in fruit softening in tomato (Wang et al., 2021b). In contrast, MaDof23 might inhibit fruit softening, as MaDof23 suppressed the expression of ripening-related genes in banana (Feng et al., 2016). Thus, distinct members of the Dof family may play divergent roles in fruit softening. However, few studies have performed a comprehensive investigation of the Dof family during fruit softening, and the molecular mechanism by which Dofs regulate fruit softening remains poorly understood.

Plant hormones play essential roles in fruit development and ripening. Of all phytohormones, abscisic acid (ABA) is considered to be a critical activator for the ripening of nonclimacteric fruits. ABA-induced fruit ripening has been described in various fruits, such as strawberry (Luo et al., 2020), grape (*Vitis vinifera*; Pilati et al., 2017), and winter jujube (*Ziziphus jujuba*; Kou et al., 2019). ABA levels gradually rise throughout development and ripening, reaching their highest levels during the ripe stage (Symons et al., 2012). The key ABA biosynthetic enzyme is 9-cis-epoxycarotenoid dioxygenase (NCED). The suppression of the gene encoding this enzyme affects ABA concentrations and fruit ripening (Sun et al., 2012). So far, how *NCED* expression is regulated to produce the necessary ABA levels during fruit ripening has not been resolved. In addition, ABA also contributes to fruit softening. For instance, ABA regulates the expression of genes encoding cell wall-degrading enzymes like rhamnogalacturonate lyase1 (FaRGL1), β-xylosidase1 (FaXyl1), and FaXTH1, thus inducing fruit softening of strawberry fruits (Molina-Hidalgo et al., 2013; Bustamante et al., 2009; Nardi et al., 2014). In bilberry (*Vaccinium myrtillus*), exogenous application of ABA raises the expression levels of *VmPL*, *VmRGLyase*, *β*-*galactosidase 1* (*Vmβ-GAL1*), *Vmβ-GAL2*, *VmXTH*, *VmCEL*, and *VmEXP1/2/3* and accelerates fruit softening (Karppinen et al., 2018). However, the TFs mediating the ABA responses and directly regulating the downstream cell wall-related genes are poorly understood.

In this study, we analyzed two sweet cherry varieties with different fruit firmness properties by transcriptome analyses, which showed that hemicellulose and pectin are crucial for maintaining cell wall integrity and fruit softening. Furthermore, we identified an ABA-mediated transcriptional cascade that controls the expression of cell wall modification-related genes, thus regulating fruit softening. We report here that the C_2_C_2_-type TFs PavDof2/6/15 bind directly to the promoters of structural genes. We also discovered an auxin response factor, PavARF8, that regulates the promoter activity of *PavDof2/15* and *PavNCED1*, participating in ABA-mediated fruit softening. Overall, our study provides insights into the molecular mechanism of fruit softening and establishes a foundation for quality improvement in sweet cherry.

## Results

### The degree of cell wall degradation during fruit softening determines the flesh type of sweet cherry fruits

**Figure 1:**
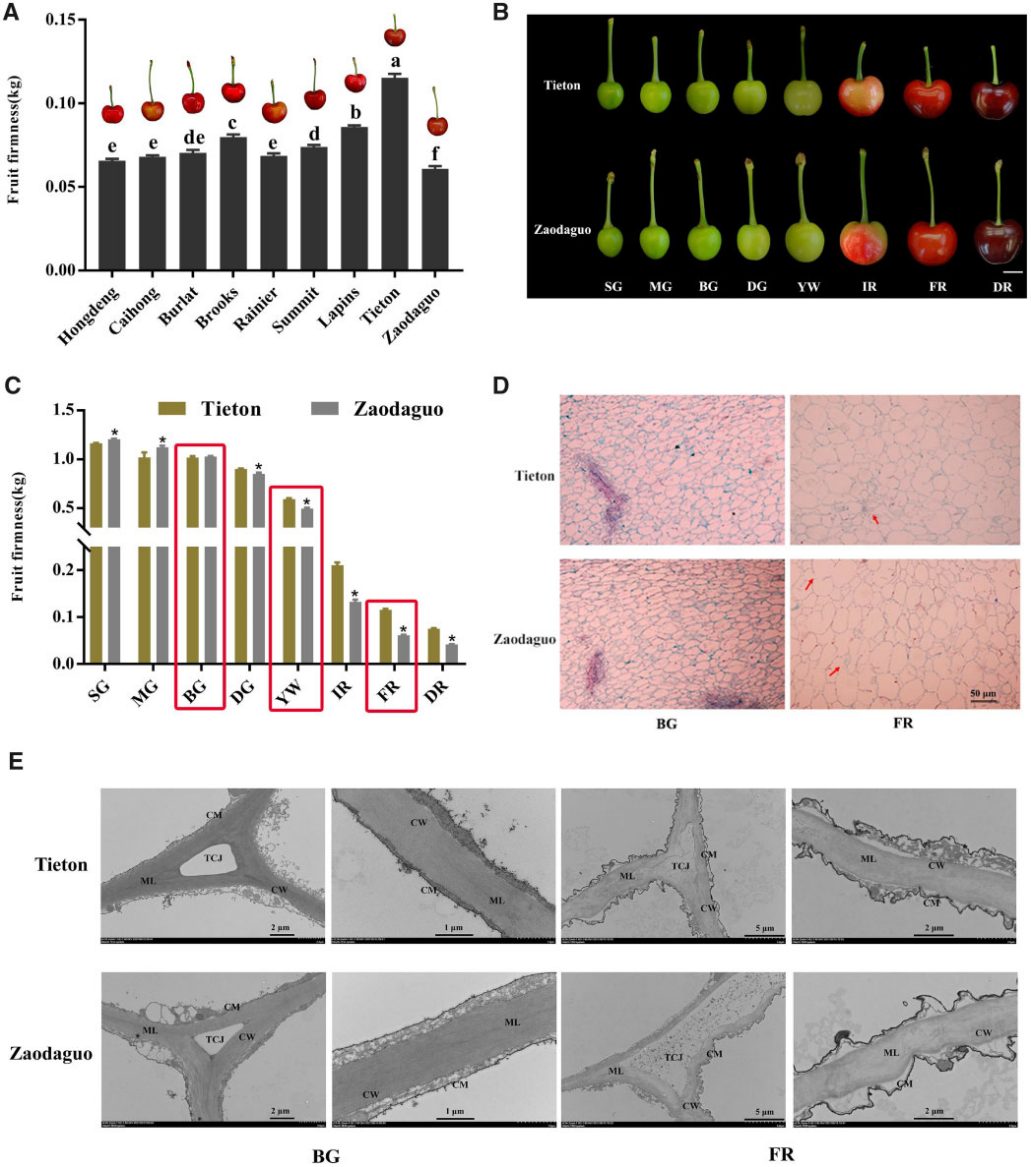
Evaluation of firmness and microstructure of cherry fruits. A, Comparison of fruit firmness in nine cherry varieties at the FR stage. B, Fruit appearance during fruit development for the varieties Tieton and Zaodaguo. The fruits have been digitally extracted for comparison. Scale bar = 1 cm. Fruit development was divided into eight periods according to Shen et al. (2014). C, Dynamic changes of firmness during fruit development in the varieties Tieton and Zaodaguo. Red rectangles represent the developmental periods selected for subsequent analysis. D, Paraffin sections of fruit flesh. The red arrows indicate the differences between the two varieties. Scale bar = 50 μm. E, Ultramicroscopy inspection of changes in the structure of cell walls in cherry fruits. CW, cell wall; CM, cell membrane; ML, middle lamella; TCJ, tricellular junction. Sections were isolated from the same location in all fruits. In A and C, data are shown as means ± sd of three biological replicates. Different letters in A indicate significant differences by Duncan’s multiple range test with *P *<* *0.05. Asterisks in C indicate statistically significant difference between the two varieties by Student’s *t* test with *P *<* *0.05.

We selected nine cherry varieties with different firmness properties to measure fruit firmness at the full-red (FR) fruit maturity stage. The variety “Tieton” had the firmest fruits, followed by “Lapins” and “Brooks”, while the variety with the softest fruits was “Zaodaguo” (Figure 1A). Then we focused on the extreme varieties Tieton and Zaodaguo for further analysis. Accordingly, we collected fruits from both varieties at eight fruit developmental stages to measure firmness (Shen et al., 2014; Figure 1B). As shown in Figure 1C, during the early stages, both varieties displayed high levels of fruit firmness. With fruit ripening, firmness gradually decreased, reaching values at the yellow white (YW) stage about half those of the small green (SG) stage, indicating that the YW period is a key period between cherry fruit ripening and softening. Notably, the firmness of Zaodaguo fruits decreased faster than Tieton fruits after the YW stage.

We also inspected the cytological characteristics of fruit in these two varieties in paraffin sections. We observed similar cellular structures for the two varieties during early big green (BG) stage, which was characterized by small and intact cell arrangement (Figure 1D). As fruit matures, the cellular volume expands concomitantly with an increase of the intercellular space due to cell wall and middle lamella degradation. Notably, compared to the compact cell structure at the FR stage seen in Tieton, we noticed numerous discontinuities in the cell wall in variety Zaodaguo, indicating that the maintenance of cell wall integrity is compromised. We further analyzed the ultrastructure of cell wall disassembly using transmission electron microscopy (TEM). The fruits of both varieties displayed an intact cell wall structure with densely arranged microfilaments, with the cell membrane adjoining the cell wall at BG period (Figure 1E). At the late developmental FR stage, cell wall integrity was disrupted, as evidenced by light-colored and loosely arranged microfilaments, with degradation of the middle lamella and plasmolysis in both Tieton and Zaodaguo fruits. We noticed that cell wall disruption is more severe in Zaodaguo than in Tieton, as the tricellular junction had fully disappeared in Zaodaguo, but remained in Tieton.

To complement the TEM analysis, we measured the changes in cell wall components across BG, YW, and FR stages in both varieties. We determined that the contents of cellulose, hemicellulose, and covalent-soluble pectin (CSP) decrease in both varieties during fruit softening, while water-soluble pectin (WSP) and ionic-soluble pectin (ISP) levels increased over the same period (Table 1). We also detected clear differences between varieties, as hard-fleshed Tieton fruits contained more hemicellulose and CSP, and less WSP and ISP, than the soft-fleshed Zaodaguo fruits at FR stage. However, the decrease in cellulose contents was comparable between the two varieties. Collectively, these findings suggest that fruit softening in sweet cherry depends on the degradation of cell wall and that differences in fruit firmness between varieties are caused by alterations of hemicellulose and pectin contents rather than cellulose.

**Table 1:** Contents of cell wall materials in the fruits of the cultivars Tieton and Zaodaguo at three different developmental periods.

Cultivar	Developmental period	CSP (mg/100 mg AIR)	WSP (mg/100 mg AIR)	ISP (mg/100 mg AIR)	Cellulose (mg/100 mg AIR)	Hemicellulose (mg/100 mg AIR)
	BG	4.41 ± 0.33^a^	1.07 ± 0.12^d^	1.02 ± 0.33^c^	16.36 ± 1.5^a^	13.42 ± 0.69^a^
Tieton	YW	3.67 ± 0.24^a^	3.22 ± 0.57^c^	2.76 ± 0.78^b^	12.62 ± 0.25^b^	8.43 ± 0.45^b^
	FR	2.39 ± 0.32^b^	5.23 ± 0.51^b^	3.49 ± 0.73^b^	12.19 ± 0.49^b^	7.39 ± 0.69^b^
	BG	4.31 ± 0.32^a^	1.02 ± 0.32^d^	1.02 ± 0.16^c^	16.65 ± 0.85^a^	12.63 ± 1.14^a^
Zaodaguo	YW	3.40 ± 0.24^a^	3.12 ± 0.24^c^	2.94 ± 0.57^b^	13.34 ± 0.76^b^	7.64 ± 0.79^b^
	FR	1.02 ± 0.16^c^	7.88 ± 0.32^a^	5.78 ± 0.24^a^	12.05 ± 0.38^b^	4.52 ± 0.43^c^

AIR, alcohol-insoluble residue. Values are means ± sd from three biological replicates. Different letters indicate significant differences, as determined by Duncan’s multiple range test with *P *<* *0.05.

### Transcriptome analysis of the hard-fleshed Tieton and soft-fleshed Zaodaguo cultivars during fruit softening

To identify the transcript changes that take place during sweet cherry fruit development, transcriptome analysis was performed using BG, YW, and FR fruits from two varieties.

**Figure 2:**
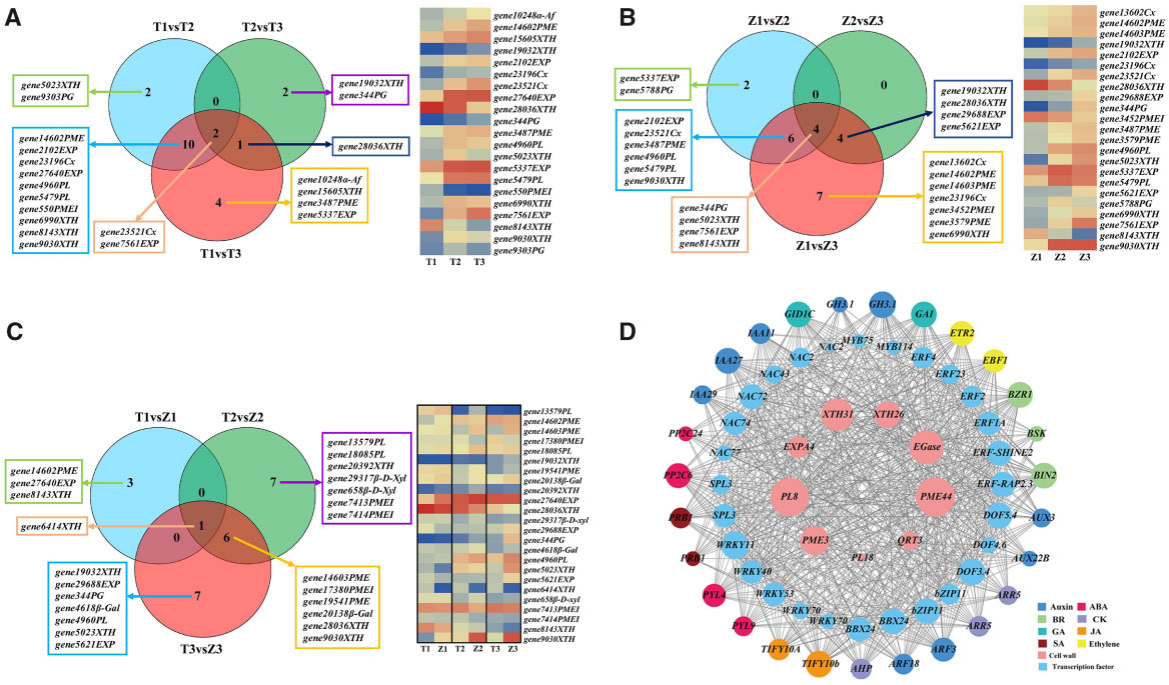
Differentially expressed structural genes identified in sweet cherry and correlation analysis of vital structural genes, TF genes, and plant hormone signaling genes. A, B, Venn diagrams of DEGs (left) and heatmap representation of expression levels (right) of structural genes for comparisons between different developmental periods in Tieton and Zaodaguo samples (comparison 1). C, Venn diagram of DEGs (left) and heatmap representation of expression levels (right) of structural genes for comparisons between Tieton and Zaodaguo at three developmental periods (comparison 2). For (A–C), the heatmaps show expression levels as Log_2_(TPM + 1). T, Tieton samples; Z, Zaodaguo samples; 1, BG stage; 2, YW stage; 3, FR stage. The gene name represents gene ID in transcriptome with its gene family. D, Co-expression analysis of structural genes, TF genes, and plant hormone signaling genes. The area of each circle refers to the number of DEGs co-expressed with each of the genes shown. The meaning of the gene acronyms is given in Supplemental Table S6. BR, brassinosteroid; CK, cytokinin; GA, gibberellic acid; JA, jasmonic acid; SA, salicylic acid.

Hierarchical clustering and principal component analysis (PCA) highlighted the excellent reproducibility of the three replicates for the same development stage (Supplemental Figure S1, A and B). The differentially expressed genes (DEGs; both upregulated and downregulated) were obtained by comparisons within inter-varieties (T1 versus T2, T2 versus T3, T1 versus T3 and Z1 versus Z2, Z2 versus Z3, Z1 versus Z3; comparison 1) and intra-varieties in different stages (T1 versus Z1, T2 versus Z2, and T3 versus Z3; comparison 2). In comparison 1, a total of 6,142 and 6,418 DEGs were identified in Tieton and Zaodaguo, respectively (Supplemental Figure S2). The number of downregulated genes was substantially higher than that of upregulated genes in both varieties. In comparison 2, 1,607 (T1 versus Z1), 2,275 (T2 versus Z2), and 2,560 (T3 versus Z3) DEGs were identified, with the increasing number of DEGs at the later stages (Supplemental Figure S2). Analysis of cell wall-related genes in Tieton and Zaodaguo identified 21 and 23 DEGs (comparison 1), respectively, whose substrates were cellulose, hemicellulose, and pectin (Figure 2, A and B; Supplemental Tables S1 and S2). In contrast, the substrates of the 24 cell wall-related DEGs identified from an intra-variety comparison (comparison 2) were mainly hemicellulose and pectin, further confirming the crucial role of hemicellulose and pectin metabolism for fruit softening (Figure 2C; Supplemental Table S3).

Next, we analyzed the DEGs of TFs using the PlantTFDB tool (Supplemental Table S4). We thus identified 172 and 169 TF genes belonging to 29 families in Tieton and Zaodaguo, respectively (Supplemental Figure S3, A, B, and D). We also identified 70 TF genes belonging to 20 families and showing differential expression between the two varieties (Supplemental Figure S3, C and E). We took a closer look at DEGs related to plant hormone signaling, which revealed 43 genes associated with eight phytohormones (Supplemental Figure S4, A–C). Next, the co-expression analysis showed that nine key structural genes including *PG*, *PL*, *PME*, and *XTH* are associated with 28 TF genes and 27 phytohormone-related genes, indicating that the phytohormone-mediated transcriptional network plays a critical role in sweet cherry fruit softening (Figure 2D; Supplemental Table S5). We validated RNA-sequencing (RNA-seq) results by reverse transcription–quantitative polymerase chain reaction (RT–qPCR), which illustrated the congruency between the two methods (Supplemental Figure S5).

### ABA promotes fruit ripening and softening in sweet cherry

**Figure 3:**
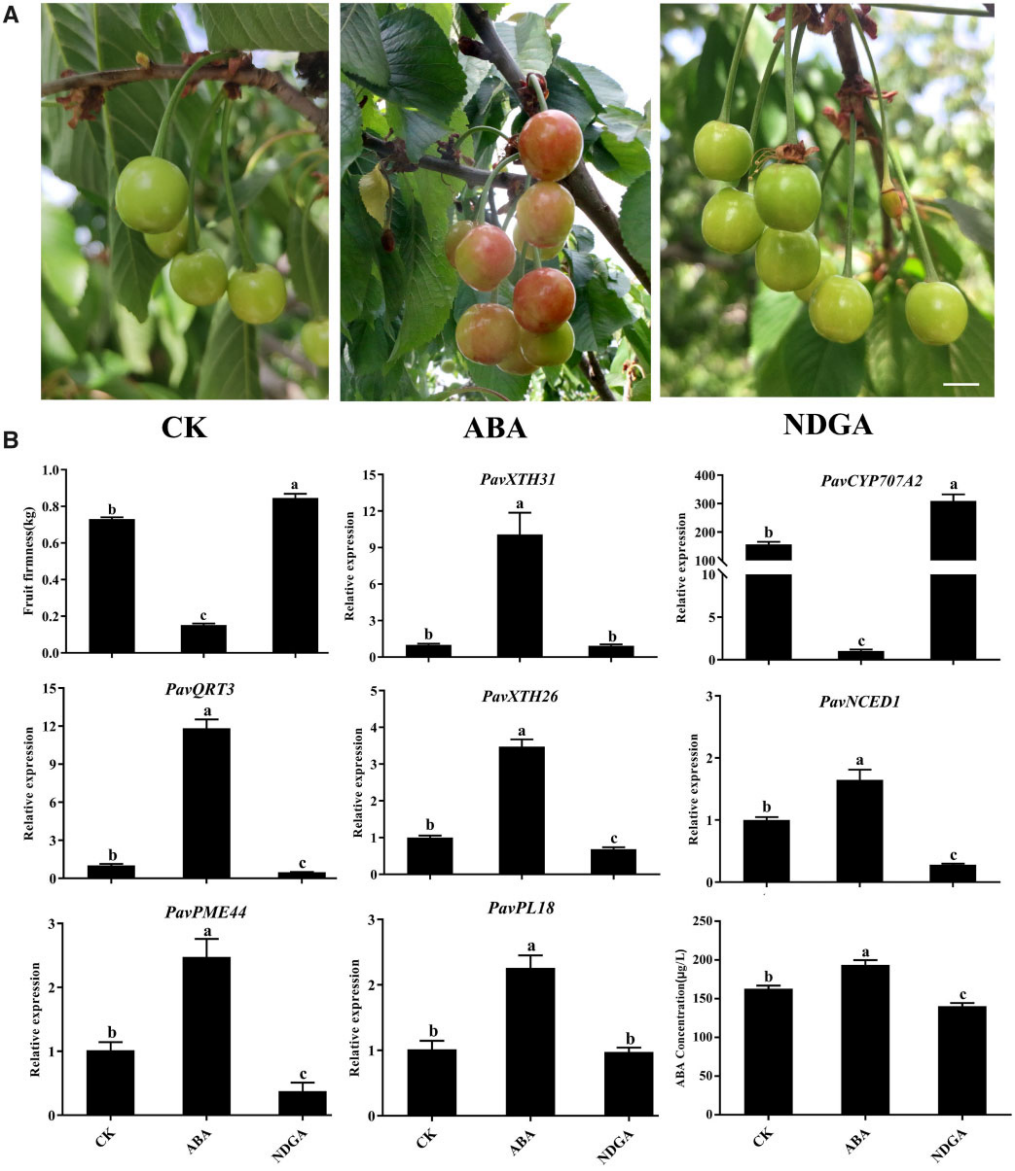
Effects of ABA treatment on fruit softening in sweet cherry. A, Phenotype of cherry fruits on the sixth day after treatments with ABA or the ABA biosynthesis inhibitor NDGA. Scale bar = 1 cm. CK, control check. B, Changes in firmness, expression of candidate structural genes and key genes related to ABA metabolism, and ABA concentrations after ABA or NDGA treatment. Data are shown as means ± sd of three biological replicates. Different letters indicate significant differences by Duncan’s multiple range test with *P *<* *0.05.

To investigate the role of ABA in regulating fruit softening, we treated cherry fruits before the hardstone stage with exogenous ABA or its biosynthesis inhibitor nordihydroguaiaretic acid (NDGA). ABA application accelerated the fruit ripening and significantly decreased fruit firmness whereas NDGA resulted in the opposite effect compared to control fruits treated with the distilled water (*P *<* *0.05, Figure 3, A and B). In agreement with this observation, the expression of five key structural genes identified from the co-expression analysis (Figure 2D) were all upregulated by exogenous ABA. In addition, ABA and NDGA treatments altered endogenous ABA levels in opposite directions, prompting us to speculate that ABA biosynthesis may be affected during ripening progression. To test this idea, we analyzed the expression levels of all *NCED* family members in the RNA-seq data, and we discovered that *PavNCED1* expression increases with fruit ripening, indicating a possible role for this gene in ABA-mediated fruit softening (Supplemental Figure S6, A and B). In addition, the expression of *PavCYP707A2*, encoding a cytochrome P450 monooxygenase responsible for ABA catabolism, decreased in response to exogenous ABA treatment, in agreement with a previous report (Li et al., 2015). These results suggest that cell wall modifications leading to fruit softening in sweet cherry might be modulated by ABA-mediated transcriptional regulation. The C_2_C_2_-type TFs PavDof2/6/15 bind to the promoters of structural genes related to cell wall modifications and regulate their transcriptional output.

**Figure 4:**
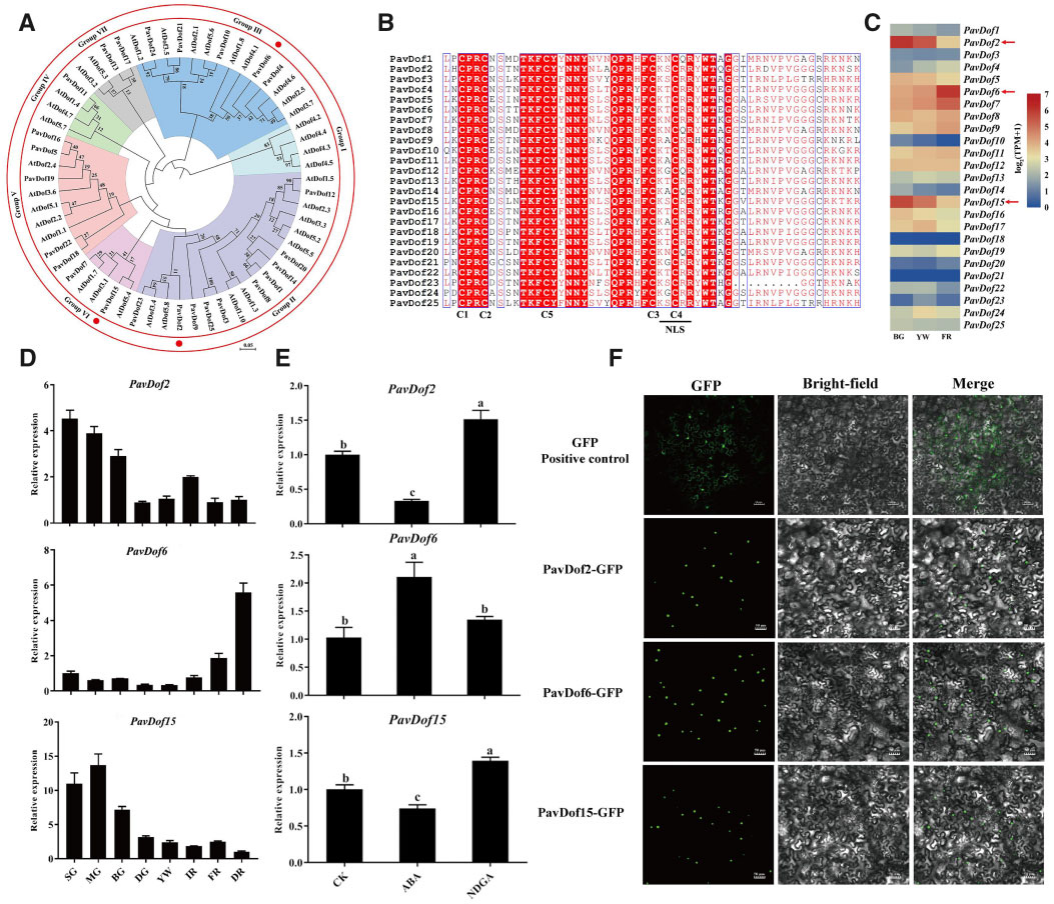
Characterization of PavDof2/6/15*.* A, Phylogenetic relationships between Dof family members in sweet cherry and Arabidopsis. The circles indicate candidate PavDofs. B, Multiple sequence alignment of the conserved Dof domain in 25 PavDof proteins. The four cysteine residues involved in the zinc finger structure and the nuclear localization signal motif are shown. Identical amino acids are highlighted. C, Heatmap representation of *PavDof* transcript levels in Zaodaguo. The arrows indicate the candidate PavDofs. D, Relative expression levels of *PavDof2/6/15* during fruit development and ripening in Zaodaguo, as determined by RT–qPCR. Data are shown as means ± sd from three biological replicates, each biological replicate contains 15 fruits. SG, big green; MG, mid green; BG, big green; DG, degreening; YW, yellow white; IR, initial red; FR, full red; DR, dark red. E, Effect of ABA treatment on *PavDof2/6/15* expression levels. *PavActin* (Genbank: FJ560908) was used as an internal control. Data are shown as means ± sd of three biological replicates. Different letters indicate significant differences by Duncan’s multiple range test with *P *<* *0.05. CK, control check. F, Subcellular localization assay of PavDof2/6/15. GFP (Positive control) or the *PavDof2/6/15-GFP* constructs driven by the CaMV 35S promoter were individually transiently infiltrated into *N. benthamiana* leaves. Scale bars = 50 μm.

To explore potential TFs involved in ABA-mediated cell wall modification during the softening of cherry fruits, we focused on the promoters of five structural genes: *gene344* (*PavQRT3*), *gene3487 (PavPME44*), *gene5479* (*PavPL18*), *gene9030* (*PavXTH31*), and *gene5023* (*PavXTH26*), all key players of fruit softening in the soft-fleshed variety Zaodaguo. We determined their promoter sequences from Zaodaguo and identified numerous Dof-binding motifs (A/TAAAG; Supplemental Figure S7). Then we searched for the annotation of our transcriptome data, resulting in the identification of 25 *PavDof* genes named *PavDof1-PavDof25* based on their chromosomal locations in the reference genome (Supplemental Figure S8 and Supplemental Table S6). Phylogenetic analysis of PavDofs and Arabidopsis (*Arabidopsis thaliana*) Dofs revealed that they cluster into seven subgroups (I–VII) (Figure 4A). Sequence alignments showed that all PavDof proteins contain a highly conserved domain consisting of 50–56 amino acid residues forming a C_2_C_2_ zinc finger domain (Figure 4B), which is typical of Dof family members. We determined that *PavDof2/6/15* exhibit the highest expression levels of all 25 *PavDof*s during fruit softening in the RNA-seq dataset of Zaodaguo (Figure 4C). Additionally, co-expression analysis also highlighted their involvement as key TFs in the regulation of fruit softening (Figure 2D). Interestingly, the expression of *PavDof6* increased with fruit development and reached its highest levels at ripening, while *PavDof2* and *PavDof15* exhibited the opposite expression pattern (Figure 4D). We analyzed the subcellular localization of PavDof2/6/15 as fusions with the green fluorescent protein (GFP) in *Nicotiana benthamiana* leaf epidermal cells. All three PavDofs tested located in the nucleus (Figure 4F).

**Figure 5:**
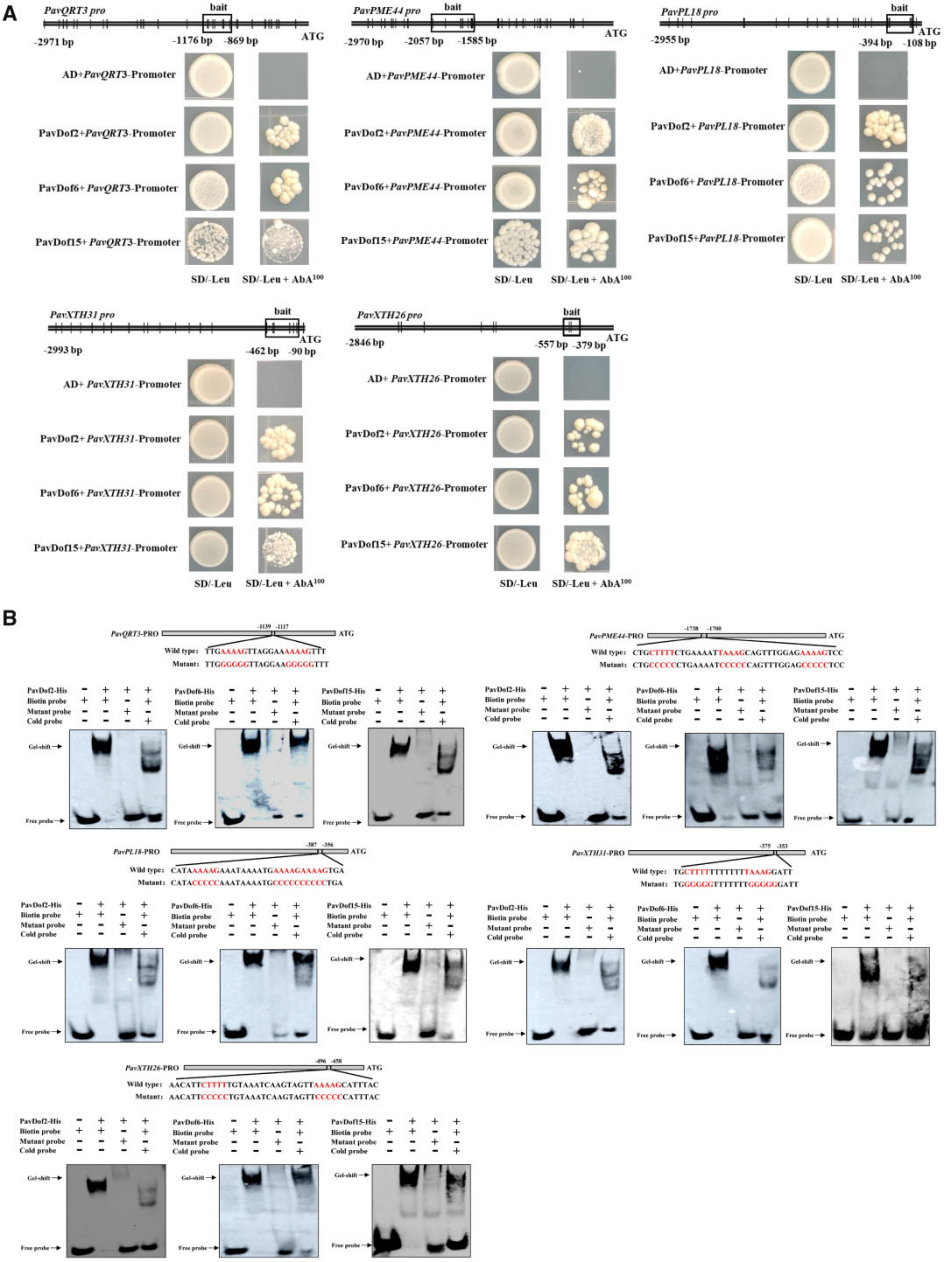
PavDof2/6/15 bind to the promoters of structural genes related to fruit softening. A, Y1H analysis of PavDof2/6/15 binding to the *PavQRT3*, *PavPME44*, *PavPL18*, *PavXTH31*, and *PavXTH26* promoters. The vertical lines indicate Dof-binding sites (A/TAAAG), and the rectangles represent the promoter fragments used for Y1H. AD, pGADT7 empty vector; SD, synthetical defined medium; AbA, aureobasidin A. B, EMSA showing the binding of PavDof2/6/15 to the *PavQRT3*, *PavPME44*, *PavPL18*, *PavXTH31*, and *PavXTH26* promoter fragments containing Dof-binding sites. Unlabeled probes were used for competition assays; − and + represent absence or presence, respectively.

**Figure 6:**
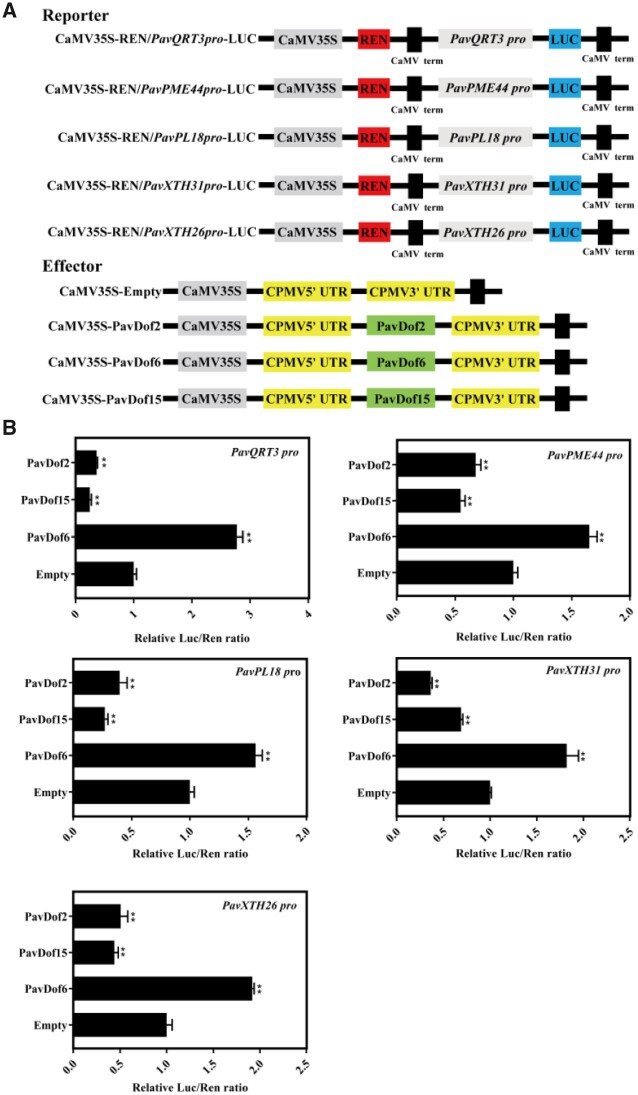
The effect of PavDof2/6/15 overexpression on the transcriptional output from the promoters of genes related to fruit softening. A, Schematic diagram of the reporter and effector constructs. The reporters consist of the *PavQRT3*, *PavPME44*, *PavPL18*, *PavXTH31*, and *PavXTH26* promoters driving the firefly luciferase (*LUC*) reporter gene, and the effectors harbour the *PavDof2/6/15* CDS driven by the CaMV 35S promoter. B, Dual-luciferase assay in *N. benthamiana* leaves showing the effects of *PavDof2/6/15* overexpression on promoter activity. Empty SK vector was used as a control. Data are shown as means ± sd from three replicates. Significant differences in values (**P <* *0.01) were determined by Student’s *t* test.

We then used yeast one-hybrid (Y1H) assays to test whether PavDof2/6/15 can directly regulate the five structural genes. As shown in Figure 5A, yeast (*Saccharomyces cerevisiae*) co-transformants harboring the prey constructs pGADT7-PavDof2/6/15 and bait constructs consisting of promoter fragments from the structural genes grew well on medium containing 100 ng · mL^−1^ aureobasidin (AbA), while the negative controls did not, suggesting that PavDof2/6/15 directly bind to the promoters of these five structural genes in yeast. We confirmed the binding of these Dof TFs by electrophoretic mobility shift assays (EMSAs). We observed a shift in mobility when recombinant purified PavDof2/6/15-HIS proteins were individually incubated with biotin probes containing the A/TAAAG motif derived from the five promoters of interest. Importantly, the shifted band decreased in intensity or even disappeared when each recombinant protein was incubated with cold probes or probes carrying mutations in the Dof-binding motif (Figure 5B). To determine the type of regulation exerted by each PavDof, we conducted a dual-luciferase activity assay. The overexpression of *PavDof6* strongly activated the promoter activities of five genes, while *PavDof2* and *PavDof15* repressed their transcriptional activity (Figure 6). Collectively, these results suggest that PavDof2/6/15 are implicated in regulating fruit softening by directly modulating the transcription of cell wall modification genes in sweet cherry.

The action of ABA on cell wall-related gene expression and, thus, fruit softening prompted us to test whether PavDofs might be involved in ABA responses. To this end, we examined the expression levels of *PavDof*s upon treatment with ABA or NDGA by RT–qPCR. We established that *PavDof2* and *PavDof15* expression levels are lower upon ABA treatment, while they were induced by NDGA. On the contrary, ABA led to a rise in relative *PavDof6* transcript levels, but NDGA had no effect (Figure 4E). Promoter analysis indicated that nearly all *PavDof*s harbor ABA-responsive elements (Supplemental Figure S9). These results suggest that ABA accelerates fruit softening partly by altering the expression *PavDof2/6/15*, whose encoding TFs in turn promote expression of cell wall modification genes.

### Transient overexpression *PavDof2/6/15* changes fruit firmness of sweet cherry

**Figure 7:**
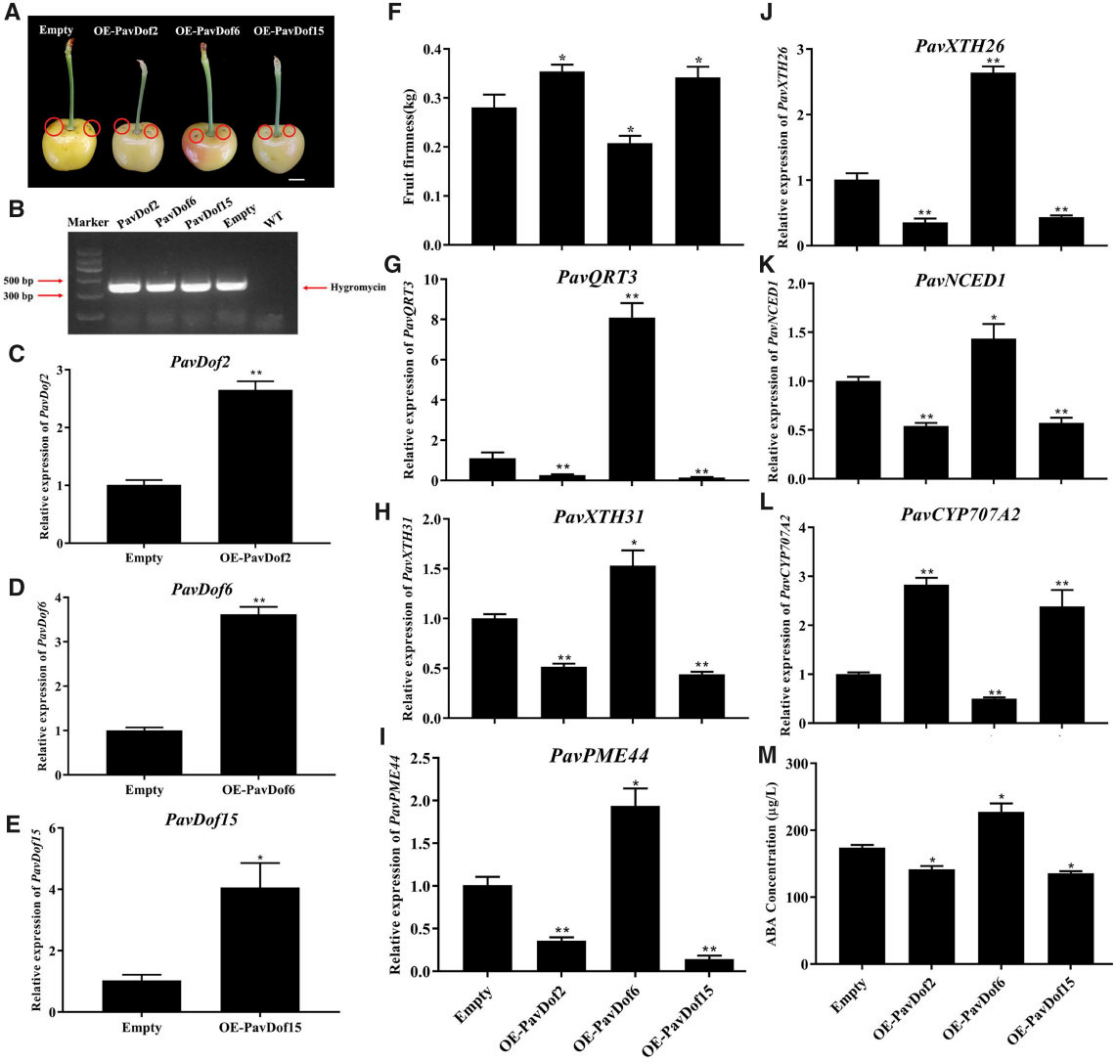
Transient overexpression of *PavDof2/6/15* in cherry fruits. A, Phenotype of transgenic cherry fruits at 2 weeks after infiltrating. The fruits have been digitally extracted for comparison. The circles indicate injection sites. Scale bar = 1 cm. B, Confirmation of transformation, based on the amplification of a 392-bp PCR product from genomic DNA extracted from transgenic fruits specific for the hygromycin resistance gene. C–M, Relative *PavDof2* (C), *PavDof6* (D), and *PavDof15* (E) expression levels, firmness (F), relative expression levels of structural genes related to fruit softening (G–J), expression of key genes related to ABA biosynthesis and metabolism (K–L), and concentrations of ABA (M) in *PavDof2*-OE, *PavDof6*-OE, *PavDof-15*-OE, and control fruits. Data are shown as means ± sd of three biological replicates, each biological replicate contains 15 fruits. Significant differences relative to empty control were determined by Student’s *t* test (**P <* *0.01, *P <* *0.05).

To ascertain the function of PavDofs, we transiently overexpressed *PavDof2/6/15* in cherry fruits in variety of “Rainier” (Figure 7A). Accordingly, we transiently infiltrated 100 fruits with a suspension of Agrobacterium (*Agrobacterium tumefaciens*) cultures harboring each transgene; we collected 45 fruits per construct for further analysis after discarding malformed fruits. To confirm the success of transient expression in cherry fruits, we used a pair of PCR primers designed to amplify a fragment of the vector from genomic DNA extracted from fruits after injecting and detected an amplicon of the right size (Figure 7B). Moreover, we determined that *PavDof2* and *PavDof6* is expressed about at 2.5- and 3.5-fold higher levels than control fruits, respectively, while *PavDof15* transcripts accumulated to levels about four-fold higher than the controls in their corresponding transgenic fruits (Figure 7, C–E). These results indicated the successful transformation of fruits with the *PavDof2/6/15* overexpression constructs. A phenotypic characterization of transgenic fruits established that fruit firmness significantly decreases in OE-*PavDof6* fruits, while it increased in OE-*PavDof2* and OE-*PavDof15* fruits (*P <* *0.05; Figure 7F), suggesting that PavDof6 promotes while PavDof2/15 represses fruit softening. We characterized the expression levels of *PavQRT3*, *PavPME44*, *PavXTH31*, and *PavXTH26* in OE-*PavDof2*, OE-*PavDof6*, and OE-*PavDof15* transgenic fruits. These cell wall-related genes were upregulated in OE-*PavDof6* transgenic fruits, but downregulated in OE-*PavDo2/f15* fruits (Figure 7, G–J). Similarly, the contents of various cell wall components were altered in OE-*PavDof2/6/15* fruits, which reflected their fruit firmness (Supplemental Figure S10). Together, these results suggest that PavDofs regulate fruit softening by modulating the expression of cell wall-related genes.

Given the different fruit firmness between Tieton and Zaodaguo, we investigated *PavDof2/6/15* expression levels in the two varieties, but observed no substantial differences (Supplemental Figure S11). A sequence analysis of the coding regions and promoters of *PavDof2/6/15* and the five key structural genes in Tieton and Zaodaguo revealed that only the *PavQRT3* promoter is different between the two varieties, with the Tieton promoter sequence missing two Dof-binding sites present in the Zaodaguo promoter sequence (Supplemental Figure S12A). PavQRT3 was highly similar to Arabidopsis QRT3, which has polygalacturonase activity and participates in cell wall degradation (Supplemental Figure S12D; Rhee et al., 2003). In agreement, the expression of *PavQRT3* continuously reached higher levels in Zaodaguo at each fruit developmental stage compared to Tieton (Supplemental Figure S12B). We tested the transcriptional activation potential of the *PavQRT3* promoter driving the *β-glucuronidase* (*GUS*) reporter gene in *N. benthamiana* leaves. We observed no differences between the Tieton and Zaodaguo *PavQRT3* promoters when infiltrated alone or together with effector constructs overexpressing *PavDof2* or *PavDof15*, which both repressed the transcriptional output of the *PavQRT3* promoter (Supplemental Figure S12C). However, *PavDof6* overexpression activated the transcription from the Zaodaguo allele of the *PavQRT3* promoter compared to the Tieton promoter (Supplemental Figure S12C). These results may thus partially explain the difference in fruit firmness between the two varieties, as explained by differential *PavQRT3* expression mediated by PavDof6.

### PavDof2/6/15 can feedback regulate the expression of *PavNCED1* to influence the ABA biosynthesis to indirectly regulate fruit softening

**Figure 8:**
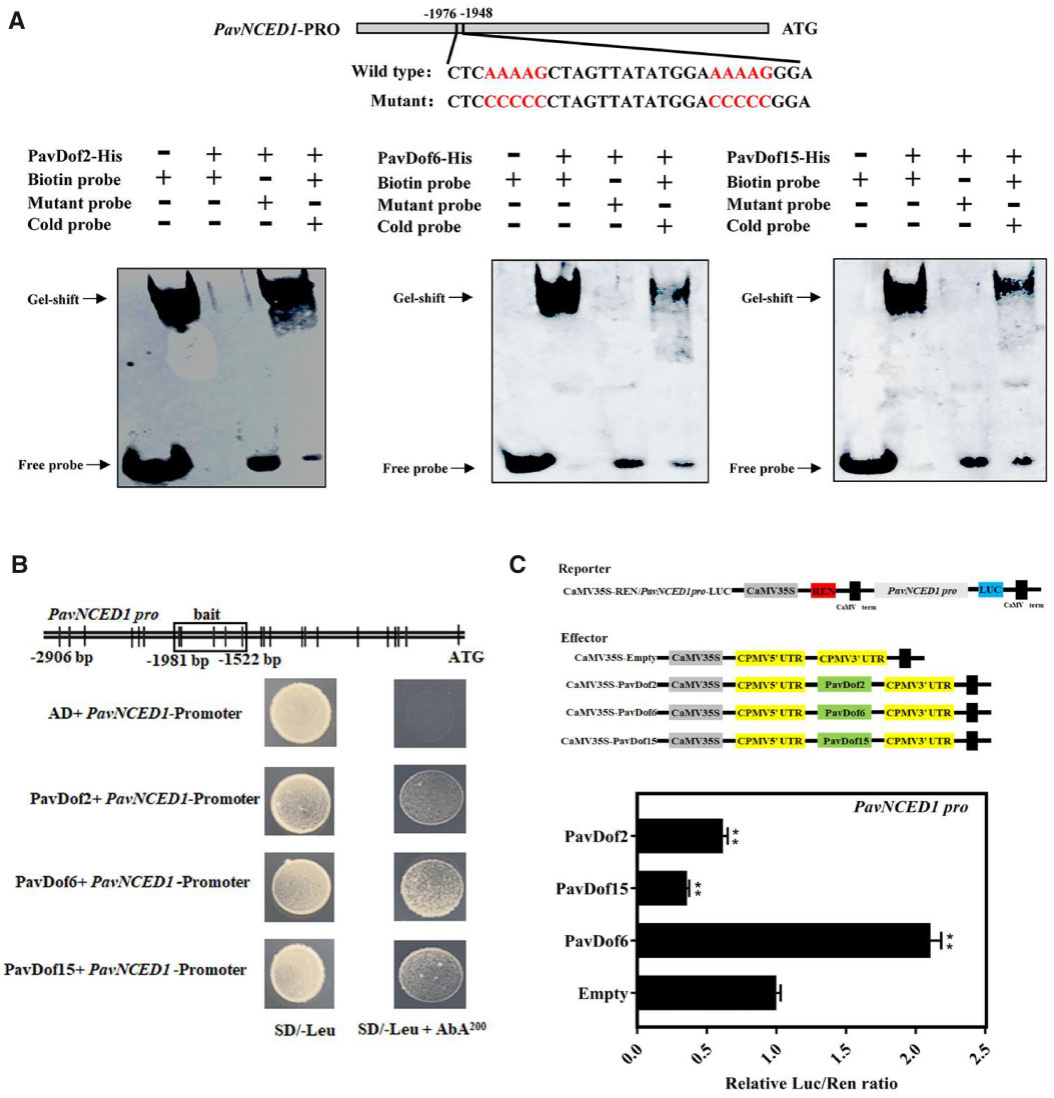
Interaction tests between PavDof2/6/15 and the *PavNCED1* promoter. A, EMSA showing that PavDof2/6/15 bind to the *PavNCED1* promoter, which contains Dof-binding sites. Unlabeled probes were used for competition assays; − and + represent absence or presence, respectively. B, Y1H analysis of PavDof2/6/15 binding to the *PavNCED1* promoter. The vertical lines indicate Dof binding sites (A/TAAAG), and the rectangles represent the promoter fragments used for Y1H. AD, pGADT7 empty vector; SD, synthetical defined medium; AbA, aureobasidin A. C, Dual-luciferase activity assay in *N. benthamiana* leaves showing the effect of *PavDof2/6/15* overexpression on the transcriptional output of the *PavNCED1* promoter. Empty SK vector was used as a control. Data are shown as means ± sd from three replicates. Significant differences relative to empty vector control were determined by Student’s *t* test (**P <* *0.01).

Due to the dramatic change in fruit firmness and cell wall-related genes expression, we speculated that ABA levels might similarly change in OE-*PavDof2*, OE-*PavDof6*, and OE-*PavDof15* transgenic fruits. To test this hypothesis, we measured the expression levels of ABA metabolism genes in transgenic fruits. Indeed, relative *PavNCED1* transcript levels increased in OE-*PavDof6* fruits and decreased in OE-*PavDof2/15* fruits compared to the control, while *PavCYP707A2* showed the opposite expression pattern (Figure 7, K–L). Consistent with these results, we observed that the ABA contents of OE-*PavDof6* transgenic fruits are higher, while they were lower in OE-*PavDof2/15* fruits compared to the control (Figure 7M). To test whether PavDofs directly regulated ABA biosynthesis, we confirmed by EMSA and Y1H assays that all three PavDofs can bind to the *PavNCED1* promoter (Figure 8, A and B). We also conducted a dual-luciferase activity assay to assess the direction of regulation imparted by PavDof proteins on *PavNCED1* transcription. Overexpression of *PavDof2* and *PavDof15* strongly repressed the promoter activities of *PavNCED1*, while PavDof6 activated its transcriptional activity (Figure 8C). Together, these observations suggest that ABA-mediated fruit softening is also subject to feedback regulation by PavDof2/6/15.

### PavARF8 acts upstream of *PavDof2/15* and participates in ABA-mediated fruit softening

**Figure 9:**
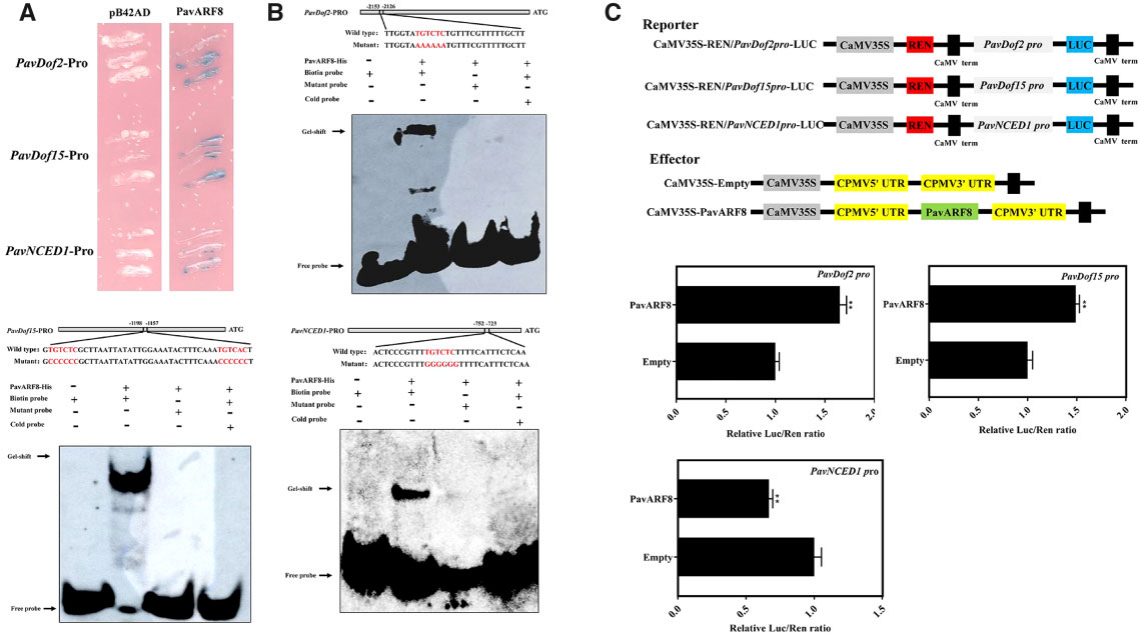
Interaction between PavARF8 and the promoters of genes related to fruit softening. A, Y1H analysis of PavARF8 binding to the *PavDof2*, *PavDof15*, and *PavNCED1* promoters. B, EMSA showing the binding of PavARF8 to *PavDof2*, *PavDof15*, and *PavNCED1* promoter fragments containing AuxREs. Unlabeled probes were used for competition assay; − and + represent absence or presence, respectively. C, Dual-luciferase activity assay in *N. benthamiana* leaves showing the effect of PavARF8 overexpression on the transcriptional output of the indicated promoters. Empty SK vector was used as a control. Data are shown as means ± sd from three replicates. Significant differences were determined by Student’s *t* test (**P <* *0.01).

To identify the potential TFs regulating the expression of *PavDof2/6/15*, we found multiple ARF-binding sites (AuxREs) in the *PavDof2/15* promoters in Zaodaguo, suggesting the involvement of ARF TFs in their transcriptional regulation (Supplemental Figure S13). We thus identified 17 *ARF* genes in our transcriptome data (Supplemental Figure S14 and Supplemental Table S7). Among them, *PavARF5* and *PavARF8* expression levels decreased during fruit development, as seen in our RNA-seq data set (Supplemental Figure S15, A and B). Exogenous ABA treatments only lead to a decrease in *PavARF8* expression, prompting us to focus on this gene (Supplemental Figure S15C). PavARF8 localized to the nucleus when a *PavARF8-GFP* construct was transiently infiltrated into *N. benthamiana* leaves (Supplemental Figure S16). Y1H assay and EMSA confirmed that PavARF8 specifically binds to the AuxREs in the *PavDof2* and *PavDof15* promoters (Figure 9, A and B). This binding activated these promoters, as evidenced by dual-luciferase assays in which *PavARF8* was transiently co-infiltrated with *LUC* reporter constructs harboring the *PavDof2* or *PavDof15* promoters (Figure 9C). In contrast, PavARF8 could directly bind to the *PavNCED1* promoter and lowered its transcriptional output (Figure 9, A–C). Together, our molecular studies demonstrate that PavARF8 acts upstream of *PavDof2/15* and participates in ABA-mediated fruit softening.

## Discussion

### The degradation rate of hemicellulose and pectin is higher in soft-fleshed Zaodaguo fruits than in hard-fleshed Tieton fruits

Fruit softening is an irreversible consequence of multiple coordinated cellular processes, especially including changes in cell wall structure and composition. The degradation of cell wall polysaccharides is thought to underlie the physiological basis of fruit softening (Fischer and Bennett, 1991; Brummell et al., 2004). Previous reports have indicated that differences in the degradation rate of cell wall components between varieties determine their fruit firmness. For example, in apples and blueberries (*Vaccinium corymbosum*), the contents of cellulose, hemicellulose, and pectin are higher in hard-fleshed varieties than in soft-fleshed varieties (Yang et al., 2018; Liu et al., 2019). In current study, analysis of the cell wall components indicated that the degradation rate of hemicellulose and pectin contributes to the different firmness between the soft-fleshed Zaodaguo and the hard-fleshed Tieton varieties (Table 1), which was not consistent with the discovery in other fruits. Moreover, transcriptome analysis showed that only structural genes that participate in hemicellulose and pectin metabolisms were enriched in the comparison between Tieton and Zaodaguo (Figure 2C). These results emphasize the distinct contribution of hemicellulose and pectin to fruit softening in sweet cherry.

### Involvement of PavDof2/6/15 in fruit softening through the regulation of genes related to cell wall modifications

Fruit softening is known to be regulated by multiple structural genes, such as *PG* (Atkinson et al., 2012), *PL* (Uluisik et al., 2016), *XTH* (Han et al., 2016), and *PME* (Xue et al., 2020). However, the general regulators that directly modulate these cell wall-related genes and thus influence sweet cherry softening are rarely studied. Dof protein are multifunctional transcription factors that influence light-responsiveness, tissue differentiation, seed development or germination, and phytohormone signaling (Zhang et al., 2018). In fact, there is limited evidence that Dof transcription factor is involved in fruit softening, notably in sweet cherry. In the present work, we functionally characterized PavDof2/6/15. We determined that PavDof2/6/15 could directly bind to the promoters of five cell wall-modifying genes by Y1H and EMSA (Figure 5). Dual-luciferase reporter assays showed that PavDof6 activated while PavDof2 and PavDof15 repressed the promoter activities of these five structural genes (Figure 6). More importantly, transient overexpression in cherry fruits further confirmed that PavDof6 was a positive regulator while PavDof2/15 was a negative regulator of cherry fruit softening through meditating the cell wall modifications and the ABA content (Figure 7). Based on the above results, we proposed that these two functions of PavDof6 and PavDof2/15 have complementary actions during sweet cherry fruit softening and jointly balance ripening.

An earlier finding indicated that several single nucleotide polymorphisms in the coding sequence (CDS) of *endo-PG* are responsible for the difference in texture between two peach (*Prunus persica* L.) varieties (Morgutti et al., 2006). In this study, we investigated sequence variation in cell wall-related genes targeted by PavDofs and identified two small deletions in the *PavQRT3* promoter from the Tieton variety, which appeared to lack two Dof-binding sites compared to Zaodaguo (Supplemental Figure S12A). Two evidences collected in this study support the notion that this insertion/deletion (InDel) polymorphism in the *PavQRT3* promoter is partly responsible for the soft-fleshed fruits characteristic of the Zaodaguo variety. First, the expression of *PavQRT3* in Zaodaguo was consistently higher than in Tieton over the course of fruit development and ripening (Supplemental Figure S12B). Second, PavDof6 activated transcription from the Zaodaguo allele of the *PavQRT3* promoter than from the Tieton *PavQRT3* promoter (Supplemental Figure S12C). Altogether, our data indicate that the InDel in Zaodaguo in part contributes to the soft texture of its fruits and that the PavDof6-*PavQRT3* module may be associated with the difference in softening between sweet cherry varieties. The precise relationship of this molecular mechanism with other varieties awaits further studies.

### Interlinked regulatory loops between ABA and PavARF8-PavDof2/15 coordinate fruit ripening and softening

ABA has long been considered a facilitator of ripening in nonclimacteric fruits (Liao et al., 2018). Previous reports have shown that ABA can accelerate fruit softening in sweet cherry (Luo et al., 2014), but the underlying mechanism remains largely unknown.

**Figure 10:**
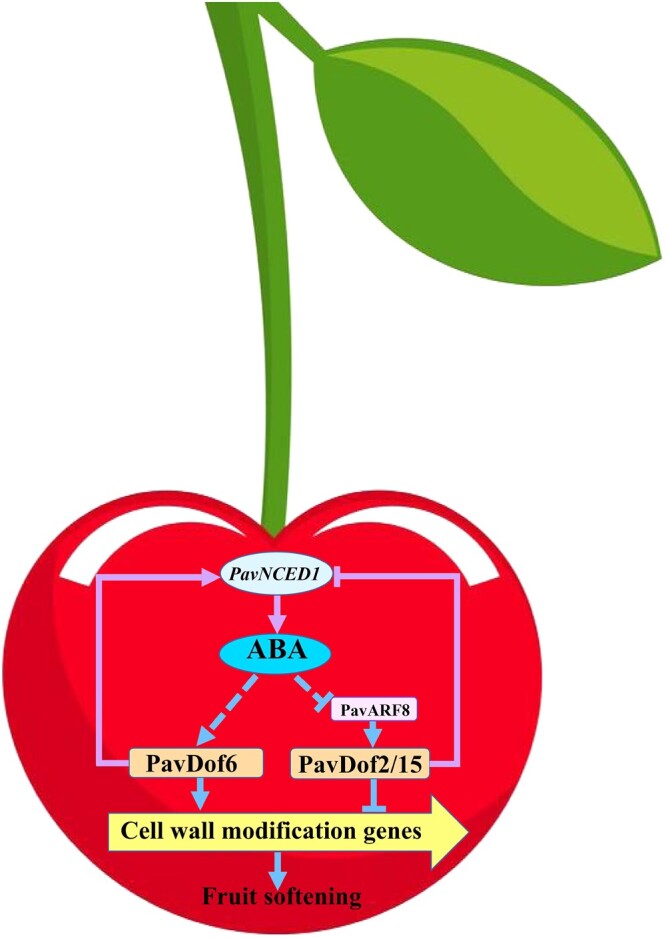
Proposed model explaining the function of PavDof2/6/15 in regulating fruit softening in sweet cherry. ABA can promote fruit softening by increasing the expression of *PavDof6* and suppressing the expression of *PavDof2/15*. PavDof2/6/15 can regulate the expression of cell wall modification genes by binding to their promoters to directly regulate fruit softening. PavDof2/6/15 can also feedback-regulate the expression of *PavNCED1* to influence ABA biosynthesis and indirectly regulate fruit softening. PavARF8 acts upstream of *PavDof2/15*, participating in ABA-mediated fruit softening. Arrows denote a positive relationship, and T-bars indicate repression.

In the typical climacteric fruit banana, the proposed model explaining the regulation of fruit softening posits that ethylene can regulate the expression of *MaBZR1/2* to mediate fruit softening, while MaBZR1/2 can also change the expression of ethylene biosynthesis genes (*1-Aminocyclopropane-1-carboxylic acid* [*ACC*] *synthase1* [*MaACS1*], *ACC oxidase13* [*MaACO13*], and *MaACO14*) and cell wall modification genes (*MaEXP2*, *MaPL2*, and *MaXET5*) to regulate fruit softening (Guo et al., 2019; Shan et al., 2020). Here, we present a similar molecular framework in non-climacteric fruits. We established that exogenous ABA treatment promotes fruit softening by regulating the expression of *PavDof2/6/15* and cell wall modification genes (Figures 3 and 4E). Moreover, PavDof2/6/15 directly regulated the transcription of cell wall modification genes and ABA biosynthesis genes simultaneously. Together, our work elucidates the molecular mechanisms of ABA-mediated fruit softening in sweet cherry. Previous studies have shown that ABA and auxin synergistically interact in some model plants and crops. For example, auxin controls seed dormancy by inducing the ARF-mediated activation of ABA-INSENSITIVE3 (ABI3), thus stimulating ABA signaling in Arabidopsis (Liu et al., 2013). ABA also regulates auxin homeostasis in rice (*Oryza sativa*) root tips to promote root hair elongation (Wang et al., 2017). However, auxin and ABA were also reported to act antagonistically to control fruit development and ripening (Liao et al., 2018), although the precise mechanism remains to be revealed. In this work, we demonstrated that the auxin response factor PavARF8 binds to the *PavNCED1* promoter (Figure 9), thus establishing a potential bridge in the crosstalk between ABA and auxin signaling during fruit softening in sweet cherry. PavARF8 also directly bound to the *PavDof2/15* promoters and activated their transcription, highlighting a potential regulatory loop between ABA signaling and the PavARF8-PavDof2/15 cascade in fruit ripening and softening in sweet cherry (Figure 10). Therefore, phytohormones and key softening regulators clearly form multiple interlocking feedback loops to maintain proper expression of cell wall modification genes and thus ensure progression towards softening. Collectively, our results provide not only the molecular basis for an in-depth mechanistic dissection of fruit ripening in sweet cherry, but also valuable clues for the broader understanding of fruit ripening in other nonclimacteric fruit species.

## Materials and methods

### Plant materials and treatments

A total of nine sweet cherry (*Prunus avium* L.) varieties including “Hongdeng”, “Caihong”, “Burlat”, “Brooks”, “Rainier”, “Summit”, “Lapins”, “Tieton”, and “Zaodaguo” were grown under field conditions at Beijing Academy of Forestry and Pomology Sciences, Beijing, China. Fruit samples at ripening period were used for firmness measurement in 2017. The hard-fleshed cherry Tieton and the soft-fleshed cherry Zaodaguo were selected for further studies. Fruit samples were collected at eight different developmental periods as previously described (Shen et al*.*, 2014): small green (SG), mid green (MG), big green (BG), degreening (DG), yellow white (YW), initial red (IR), full red (FR), and dark red (DR) at about 7, 12, 18, 24, 27, 31, 37, 46 days post anthesis (DPA) for Tieton and 7, 10, 15, 19, 22, 25, 28, 32 DPA for Zaodaguo in 2018. Fruits were sampled for their uniform size, same appearance, and no defects. Thirty fruits were analyzed to measure their firmness, and the other 30 fruits were immediately cut and frozen in liquid nitrogen and stored at −80°C for further analysis.

The variety of Zaodaguo was used for ABA and NDGA treatments. Fruits growing on the lateral branches of the trees were selected for treatments at SG period during 2018. At least three lateral branches were directly sprayed with either distilled water, 0.2-mM ABA or 0.2-mM NDGA (all solutions contained 0.1% v/v Tween-20), until droplets appeared on the surface of the fruit. The treatments were performed every 1–2 days for a total of four times. On the sixth day after treatments, samples were collected for subsequent analyses.

### Measurement of fruit firmness and phytohormones contents

The fruit firmness was determined using the TA. XT Texture Analyser (Stable Microsystems, Godalming, UK), following a method described previously (Zhai et al., 2021). The ABA content was measured with an ELISA kit following the manufacturer’s instructions (Gelatins, China).

### Microstructure assessment of cherry fruits

The paraffin section analysis was performed according to Han et al. (2016). The TEM analysis was performed as Xu et al. (2016) described.

### Extraction and measurement of the cell wall materials

The extraction of cell wall followed previous report (Zhai et al., 2021). The pectin content was measured by the m-hydroxydiphenol method (Blumenkranz and Asboe-Hansen, 1973) with galacturonic acid as a standard. The cellulose and hemicellulose contents were quantified by the anthrone method (Dische, 1962) using glucose as standards.

### Transcriptome sequencing of sweet cherry

Eighteen libraries representing the six fruit samples at periods of BG, YW and FR of Tieton and Zaodaguo were constructed. Transcriptome sequencing was performed on an Illumina HiSeq 2500 platform. The detailed analysis procedure was according to previously described (Wang et al., 2021a).

For co-expression analysis, a Pearson correlation evaluation was performed in SPSS (Version 20) software using the transcripts per million (TPM) values from all samples. An absolute Pearson correlation coefficient of ≥0.9 and a *P* ≤ 0.05 were used to identify co-expressed genes. The co-expression network was visualized by the software of Cytoscape (Version 3.7.0).

### Bioinformatics analysis of *PavDofs* in sweet cherry

The prediction of molecular weights and isoelectric points (PI), chromosomal locations, analysis of cis-elements in the promoters, phylogenetic tree construction, and multiple sequence alignment were according to Zhai et al*.* (2021). The heatmap of genes expression was visualized by TBtools (Version JRE1.6) using the TPM value in the transcriptome data.

### RNA extraction and RT–qPCR

Total RNA extraction and RT–qPCR was performed according to Guo et al. (2018). Sweet cherry *Actin 1* (GenBank accession: FJ560908) was used for normalization.

### Subcellular localization

The CDS of *PavDof2/6/15* and *PavARF8* containing BglII/SpeI restriction sites were amplified and cloned into the pCAMBIA1302 vector, which enabled *PavDof2/6/15* and *PavARF8* to be fused with GFP. Transformation was carried out as previously described (Liu et al., 2010). Fluorescence signals were observed and captured using an Olympus FluoView 3000 confocal microscope equipped with Olympus FluoView FV10-ASW 4.0 Viewer Software. GFP was excited at 488 nm and the emitted signal was captured at 500–540 nm. Images were captured using 5% of the maximum light intensity value and gain of 600–650.

### Y1H

For PavDofs, the CDS of *PavDof2/6/15* containing EcoRI/BamHI restriction sites were ligated into the pGADT7 vector. The promoters of *PavQRT3*, *PavPME44*, *PavPL18*, *PavXTH3*, *PavXTH26*, and *PavNCED1* that contained the cis-acting elements A/TAAAG were isolated from Zaodaguo and cloned into the KpnI/SalI sites of the pAbAi vector. The Y1H assay was performed according to a Matchmaker Gold Yeast One-Hybrid Library Screening System (Clontech, San Francisco, USA). For PavARF8, the CDS of *PavARF8* containing EcoRI/XhoI restriction sites was cloned into the pB42AD vector. The promoters of *PavDof2*, *PavDof15*, and *PavNCED1* that contained the AuxREs were isolated from Zaodaguo and cloned into the EcoRI/KpnI sites of the placZ vector. The Y1H assay was performed according to the method of the Matchmaker Gold Y1H system given in the Yeast Protocols Handbook, Clontech (Japan).

### EMSA

The CDS of *PavDof2/6/15* and *PavARF8* containing BamHI/SacI restriction sites were fused to the pET-30 a (+) vector to construct the His-PavDof2/6/15 and His-PavARF8 expression vector, and the recombinant plasmid were transformed into *Escherichia coli* strain BL21 (DE3). The His-PavDof2/6/15 and His-PavARF8 proteins were induced by 0.5-mM isopropyl β-d-1-thiogalactopyranoside at 16°C for 12 h, and the recombinant protein was purified by the elution of gradient imidazole-containing buffers. Based on the results of Y1H, probes containing the A/TAAAG motifs and AuxRE were synthesized and labeled with biotin by Shanghai Sangon Biotechnology (Shanghai, China), the original fragments without biotin labeling were used as a competitor. EMSA was performed according to the Light Shift Chemiluminescent EMSA Kit (Beyotime, China).

### Dual-luciferase reporter assays

The promoters of candidate genes were amplified from Zaodaguo and fused to the BamHI/NcoI sites of the reporter vector pGreen II 0800-LUC. The CDS of *PavDof2/6/15* and *PavARF8* containing BamHI/KpnI restriction sites were inserted into the effector vector pGreen II 0029 62-SK. Infiltration and promoter activity analysis were performed as previously described (Gao et al., 2018).

### *GUS* reporter gene assay

The promoters of *PavQRT3* containing HindIII/NcoI restriction sites from Tieton and Zaodaguo were cloned to the GUS gene in the pCAMBIA1301 vector. The 35S: *PavDof2/6/15* construct was used as an effector, and 35S: LUC was used as an internal control. The vector of 35S: LUC, effector, and reporter constructs were transformed into *A. tumefaciens* strain GV3101 harboring the pSoup plasmids. Infiltration and GUS activity analysis were performed as previously described (Wang et al., 2020).

### Transient overexpression in cherry fruits

The transient overexpression was performed by previously described (Fu et al., 2005), with some modifications. The CDS of *PavDof2/6/15* without the stop codon were cloned into the BglII/SpeI sites of the pCAMBIA1302 vector and introduced into *A. tumefaciens* strain EHA105. Approximately 200-μL *A. tumefaciens* suspension containing PavDof2/6/15 and the control empty vector was then separately introduced into the fruits at 20 DPA by injecting it until whole fruit was permeated. One hundred fruits were infiltrated for each gene. The inoculated fruits were collected at two weeks after infiltrating. After discarding the malformed fruits, 45 fruits were collected for subsequent analysis. The sweet cherry variety Rainier was used for this experiment. The genomic DNA was extracted using the CTAB method (Chang et al., 2007).

All primers used in this study are listed in Supplemental Table S8.

### Statistical analysis

All statistical analyses were performed by SPSS 20.0 (Windows; SPSS Inc., Chicago, IL, USA). The significance analysis was determined by the one-way ANOVA followed by Duncan’s multiple range test or Student’s *t* test*. P *<* *0.05 was considered the significant difference. *P *<* *0.01 was considered the extremely significant difference.

### Accession numbers

Sequence data from this article can be found in the NCBI website (https://www.ncbi.nlm.nih.gov/) under accession numbers PavDof2 (LOC110762675), PavDof6 (LOC110749659), PavDof15 (LOC110753378), PavARF8 (LOC110770398), PavQRT3 (LOC110749493), PavPME44 (LOC110749154), PavPL18 (LOC110751500), PavXTH31 (LOC110755396), PavXTH26 (LOC110751130), and PavNCED1 (LOC110772343). The data of RNA-seq has been deposited at NCBI Short Read Archive (SRA) database and the accession number is PRJNA814738.

## Supplemental data

The following materials are available in the online version of this article.

**Supplemental Figure S1.** Correlation and PCA of samples used in RNA-seq.

**Supplemental Figure S2.** The number of DEGs in all comparisons.

**Supplemental Figure S3.** Characterization of differentially expressed TF genes in sweet cherry.

**Supplemental Figure S4.** Differential expression of plant hormone signaling genes in sweet cherry.

**Supplemental Figure S5.** Validation of RNA-seq data by RT–qPCR.

**Supplemental Figure S6.** Expression profiles of *PavNCED* genes in sweet cherry.

**Supplemental Figure S7.** Schematic diagram of the promoters of structural gene.

**Supplemental Figure S8.** Chromosomal distribution of *PavDof* genes in sweet cherry.

**Supplemental Figure S9.** Schematic representation of predicted regulatory cis-elements in the promoters of *PavDof* family genes.

**Supplemental Figure S10.** Composition of the cell wall in transgenic cherry fruits.

**Supplemental Figure S11.** Expression profile of *Dof* genes in sweet cherry.

**Supplemental Figure S12.** Regulation of the *PavQRT3* promoter by PavDof2/6/15 from Tieton and Zaodaguo.

**Supplemental Figure S13.** Predictions of AuxREs in the promoters of *PavDof2/15* and *PavNCED1.*

**Supplemental Figure S14.** Chromosomal location of *PavARF* genes in sweet cherry.

**Supplemental Figure S15.** The expression pattern of *PavARFs* in Zaodaguo.

**Supplemental Figure S16.** Subcellular localization assay of PavARF8.

**Supplemental Table S1.** Differential expression of structural genes related to fruit softening in the comparisons of different developmental periods in Tieton.

**Supplemental Table S2.** Differential expression of structural genes related to fruit softening in the comparisons of different developmental periods in Zaodaguo.

**Supplemental Table S3.** Differential expression of structural genes related to fruit softening in the comparisons between Tieton and Zaodaguo at three developmental stages.

**Supplemental Table S4.** Predicted transcription factors.

**Supplemental Table S5.** The abbreviations of co-expressed genes.

**Supplemental Table S6.** The Dof TFs identified in sweet cherry.

**Supplemental Table S7.** The ARF TFs identified in sweet cherry.

**Supplemental Table S8.** Primers.

## Supplementary Material

kiac440_Supplementary_Data

## Notes

Z.Z., Y.L., and T.L. designed the experiments and wrote the manuscript. Y.W., Y.S., C.F., X.P., and Y.X. performed the transcriptome analysis. B.D. performed the RNA extraction and gene expression analysis. X.Zhang and X.Zhou performed the transient injection in cherry fruits. C.W. performed the phytohormone treatments. Z.Z. performed most of the remaining experiments.

The author responsible for distribution of materials integral to the findings presented in this article in accordance with the policy described in the Instructions for Authors (https://academic.oup.com/plphys/pages/general-instructions) is: Tianhong Li (lith@cau.edu.cn).
