# Supplementary material for: Abscisic acid-responsive transcription factors PavDof2/6/15 mediate fruit softening in sweet cherry
Source: Plant Physiol. 2022 Sep 21;190(4):2501–18. doi: 10.1093/plphys/kiac440 (PMC9706453; doi:10.1093/plphys/kiac440)
Supplement: kiac440_Supplementary_Data [file kiac440_supplementary_data.zip › kiac440_Supplementary_Data/Supplemental_Figures_S1_S16.pdf]

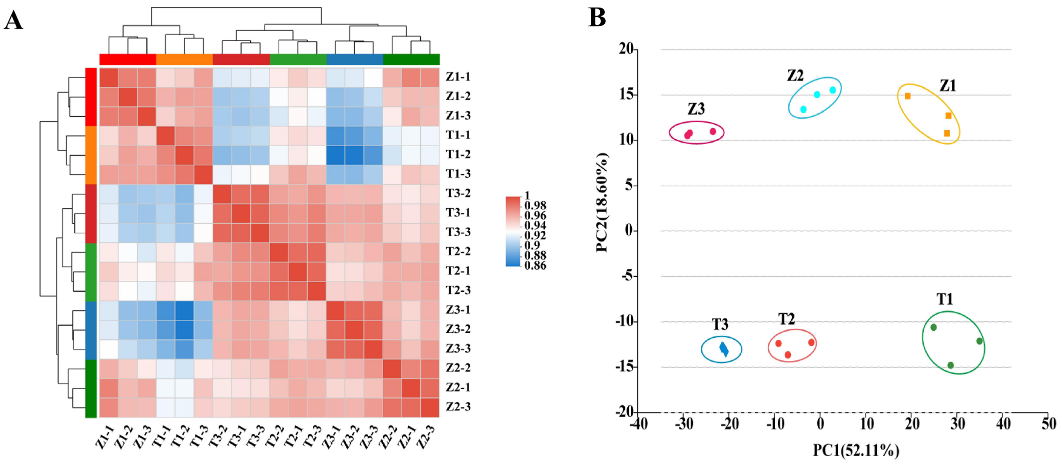

Supplemental Figure S1. Correlation and principal component analysis (PCA) of samples used in RNA-seq. A, Correlation analysis between all samples from Tieton and Zaodaguo fruits. Expression levels in TPM were used for hierarchical clustering. Each square represents the degree of correlation between samples (technical triplicates for each biological sample). **B,** PCA of the nine samples from Tieton fruits and nine samples from Zaodaguo fruits. The distance between samples represents the degree of similarity. T, Tieton; Z, Zaodaguo; 1, big green (BG); 2, yellow white (YW); 3, full red (FR).

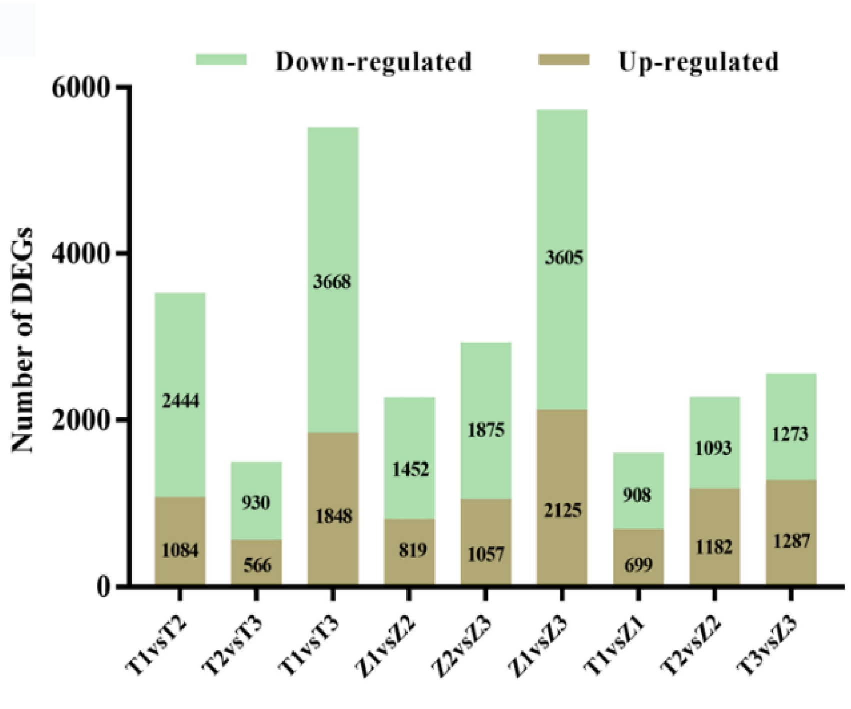

Supplemental Figure S2. The number of differentially expressed genes (DEGs) in all comparisons. T, Tieton; Z, Zaodaguo; 1, big green (BG); 2, yellow white (YW); 3, full red (FR).

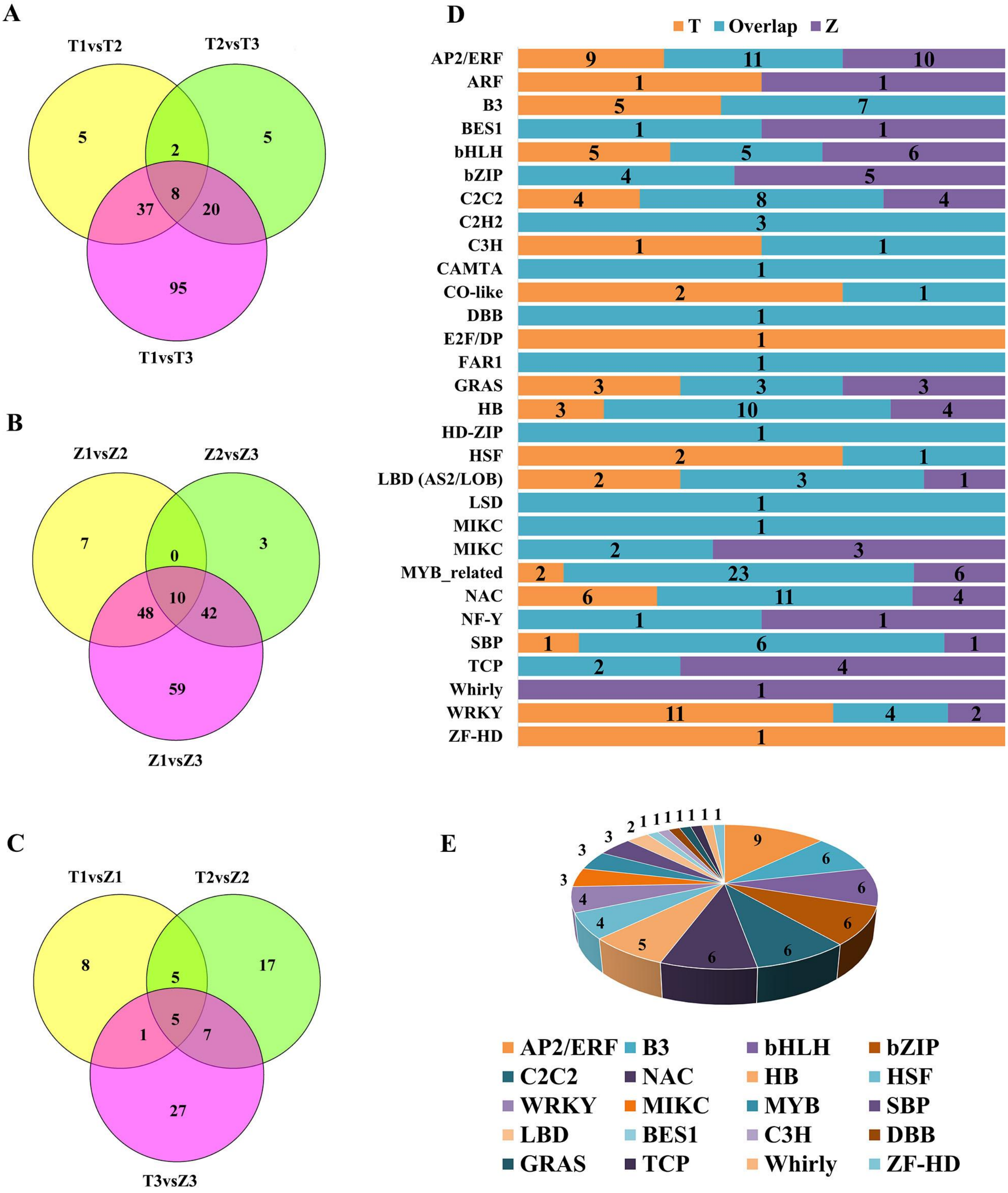

Supplemental Figure S3. Characterization of differentially expressed transcription factor (TF) genes in sweet cherry. (A-C), Venn diagrams of differentially expressed TF genes in different developmental periods in Tieton **(A)** and Zaodaguo **(B)** fruits (comparison 1) and in comparisons between the two varieties (comparison 2) **(C)**. T, Tieton; Z, Zaodaguo; 1, big green (BG); 2, yellow white (YW); 3, full red (FR). **D,** Distribution of differentially expressed TF families in comparisons between different development periods in Tieton and Zaodaguo. **(e)** Number of differentially expressed TF genes identified in comparisons between the two varieties.

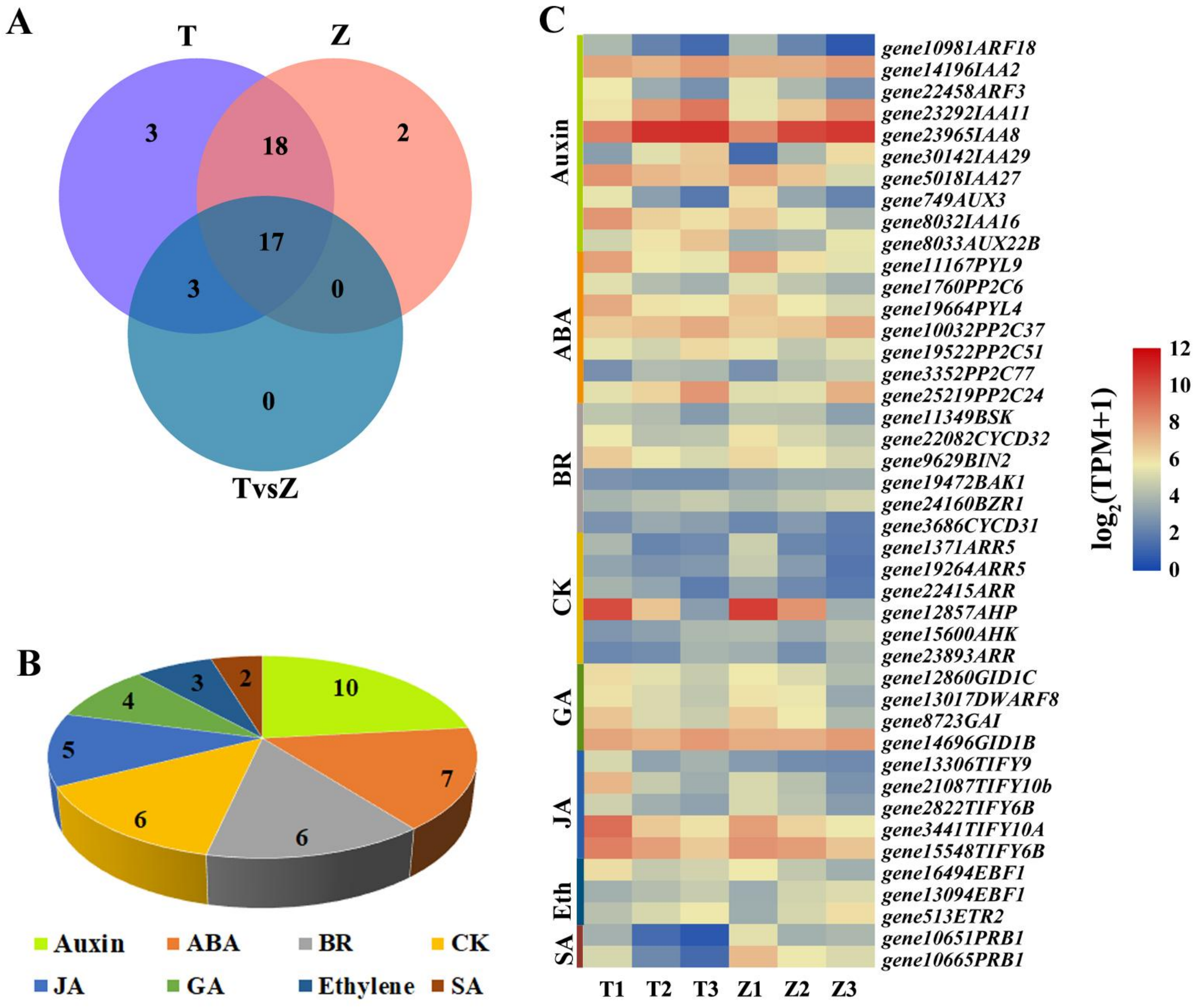

Supplemental Figure S4. Differential expression of plant hormone signalling genes in sweet cherry. A, Venn diagram showing the extent of overlap between genes identified in the indicated pairwise comparisons. T and Z refer to DEGs in comparisons between different developmental periods in Tieton and Zaodaguo (comparison 1); T vs Z refers to DEGs in comparisons between the two varieties in three developmental periods (comparison 2). **B,** Number of differentially expressed plant hormone signalling genes. **C,** Heatmap representation of DEGs related to different plant hormones. The colour scale of the heatmap represents expression levels as Log_2(TPM+1). T, Tieton; Z, Zaodaguo; 1, big green (BG); 2, yellow white (YW); 3, full red (FR). ABA, abscisic acid; BR, brassinosteroid; CK, cytokinin; GA, gibberellic acid; JA, jasmonic acid; SA, salicylic acid.

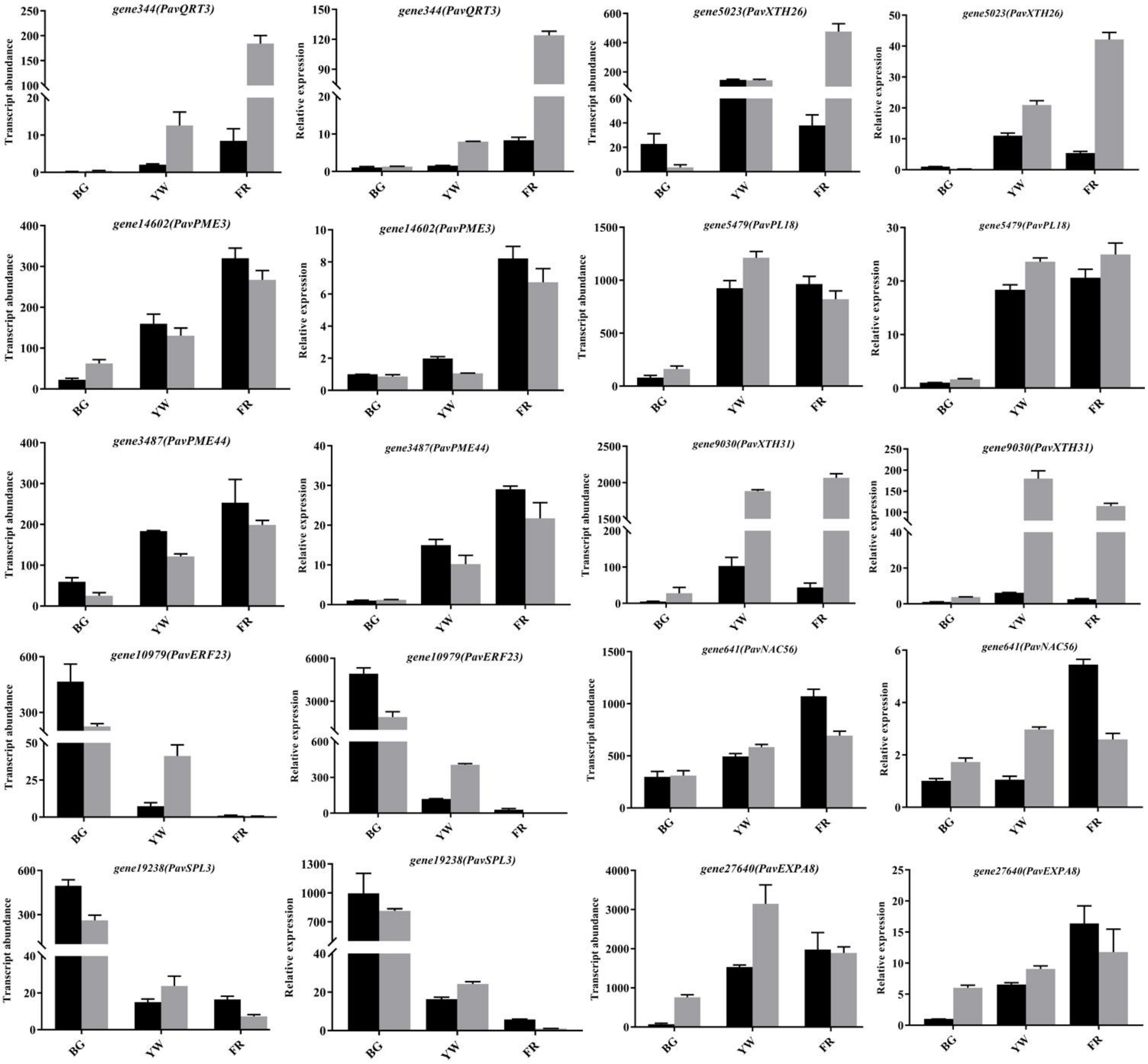

Supplemental Figure S5. Validation of RNA-seq data by RT-qPCR. Transcript levels obtained by RNA-seq are shown as TPM values in left. Relative expression determined by RT-qPCR and expressed in $2^{-\Delta\Delta}$ct is shown in right. BG, big green; YW, yellow white; FR, full red. Data are shown as means ± SD of three biological replicates. The black columns represent Tieton, the grey columns represent Zaodaguo.

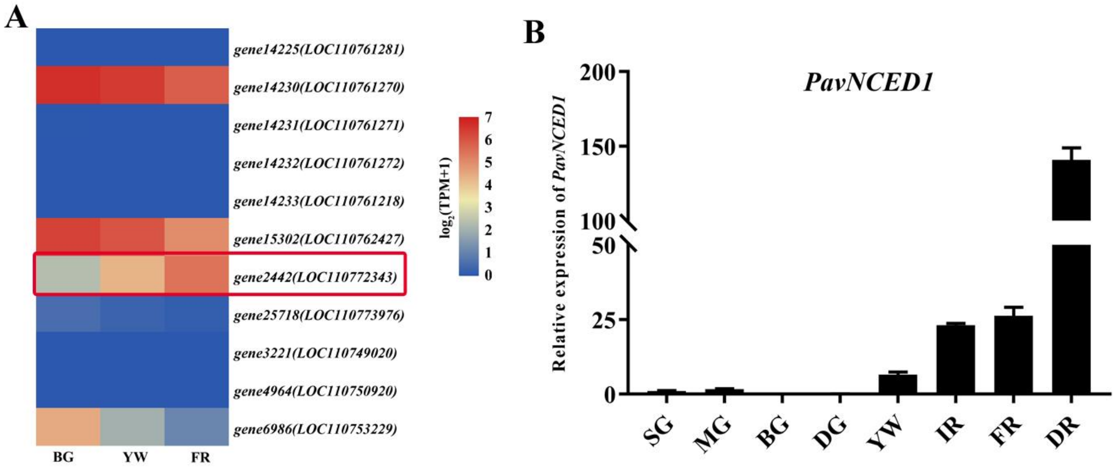

Supplemental Figure S6. Expression profiles of *PavNCED* genes in sweet cherry. A, Heatmap representation of *PavNCED* expression levels in the RNA-seq data for Zaodaguo. The red rectangle indicates the candidate *PavNCED1*. **B,** Relative *PavNCED1* transcript levels during fruit development and ripening in Zaodaguo, as determined by RT-qPCR. *PavActin* (Gene bank: FJ560908) was used as an internal control. SG, big green; MG, mid green; BG, big green; DG, degreening; YW, yellow white; IR, initial red; FR, full red; DR, dark red. Data are shown as means ± SD from three biological replicates.

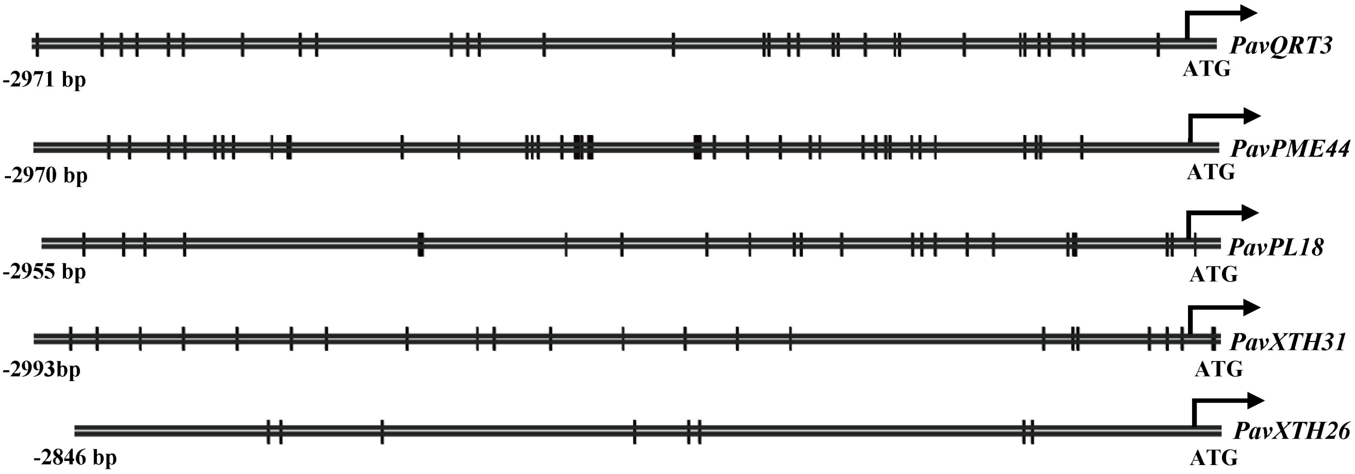

Supplemental Figure S7. Schematic diagram of the promoters of structural gene. Black vertical lines indicate *cis*-acting elements potentially associated with the DNA binding with Dof TFs.

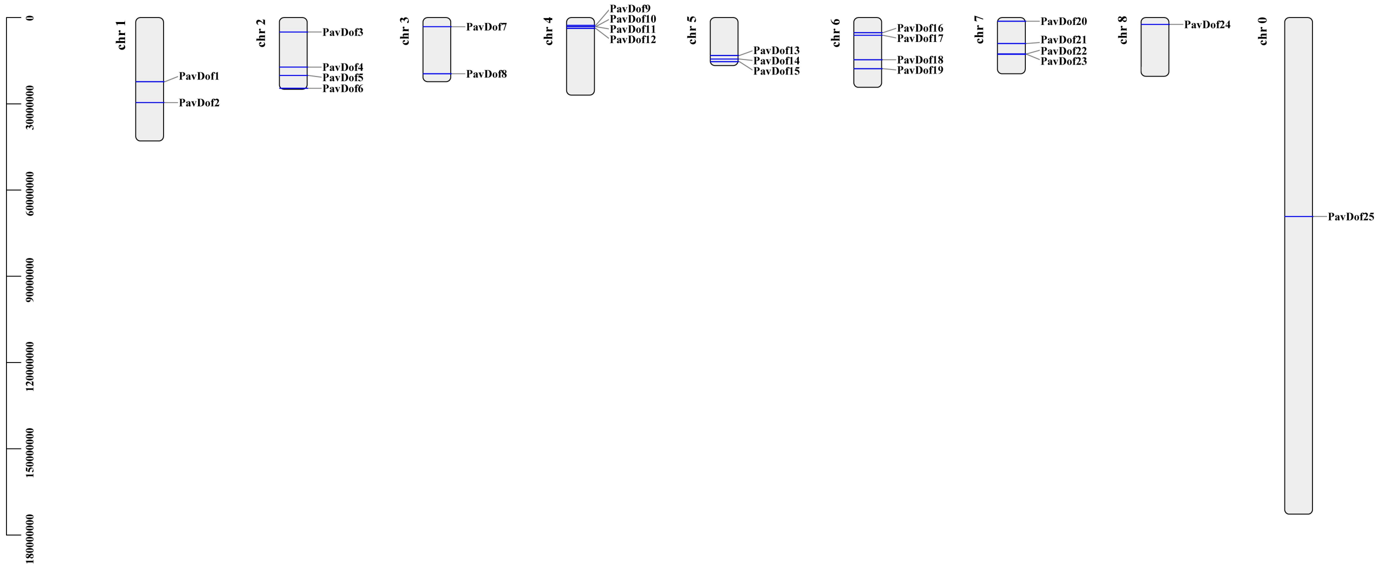

Supplemental Figure S8. Chromosomal distribution of *PavDof* genes in sweet cherry.

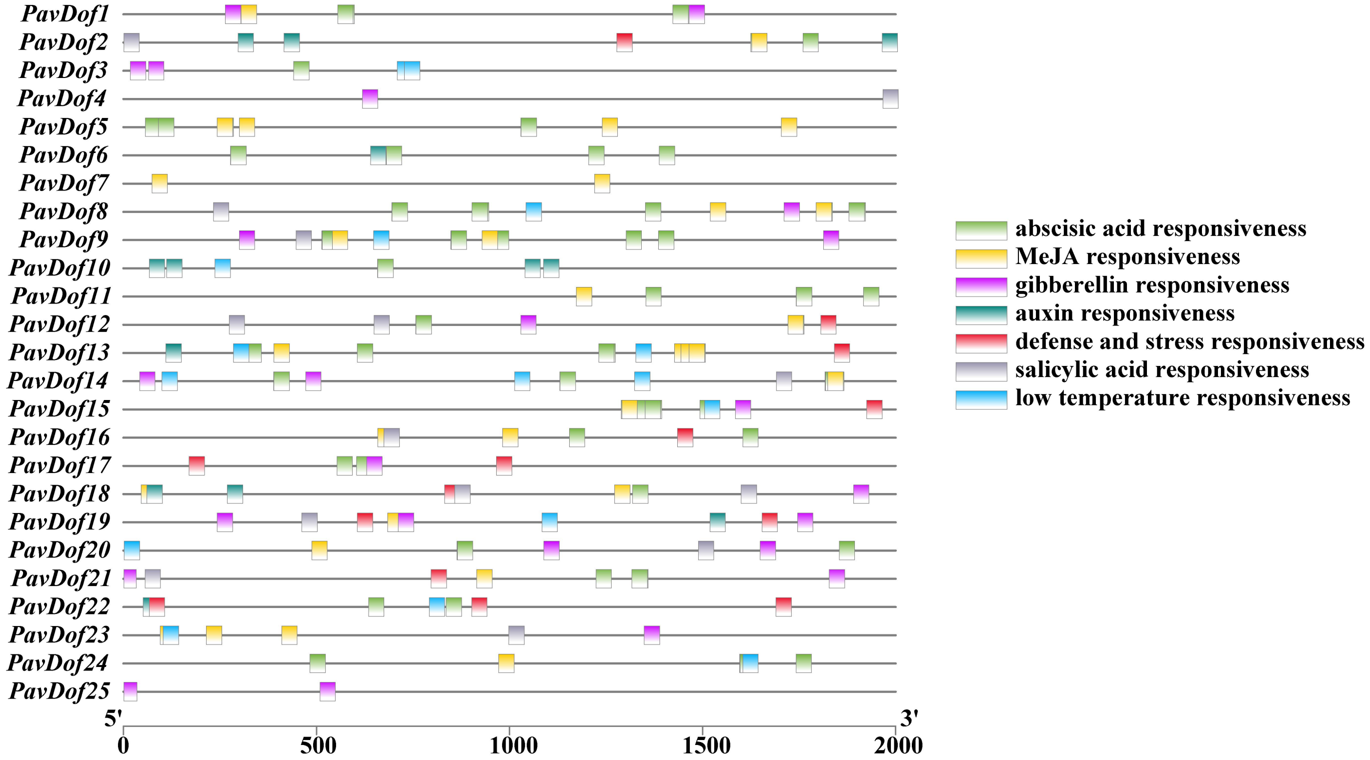

Supplemental Figure S9. Schematic representation of predicted regulatory *cis*-elements in the promoters of *PavDof* family genes. The distances represent base pairs.

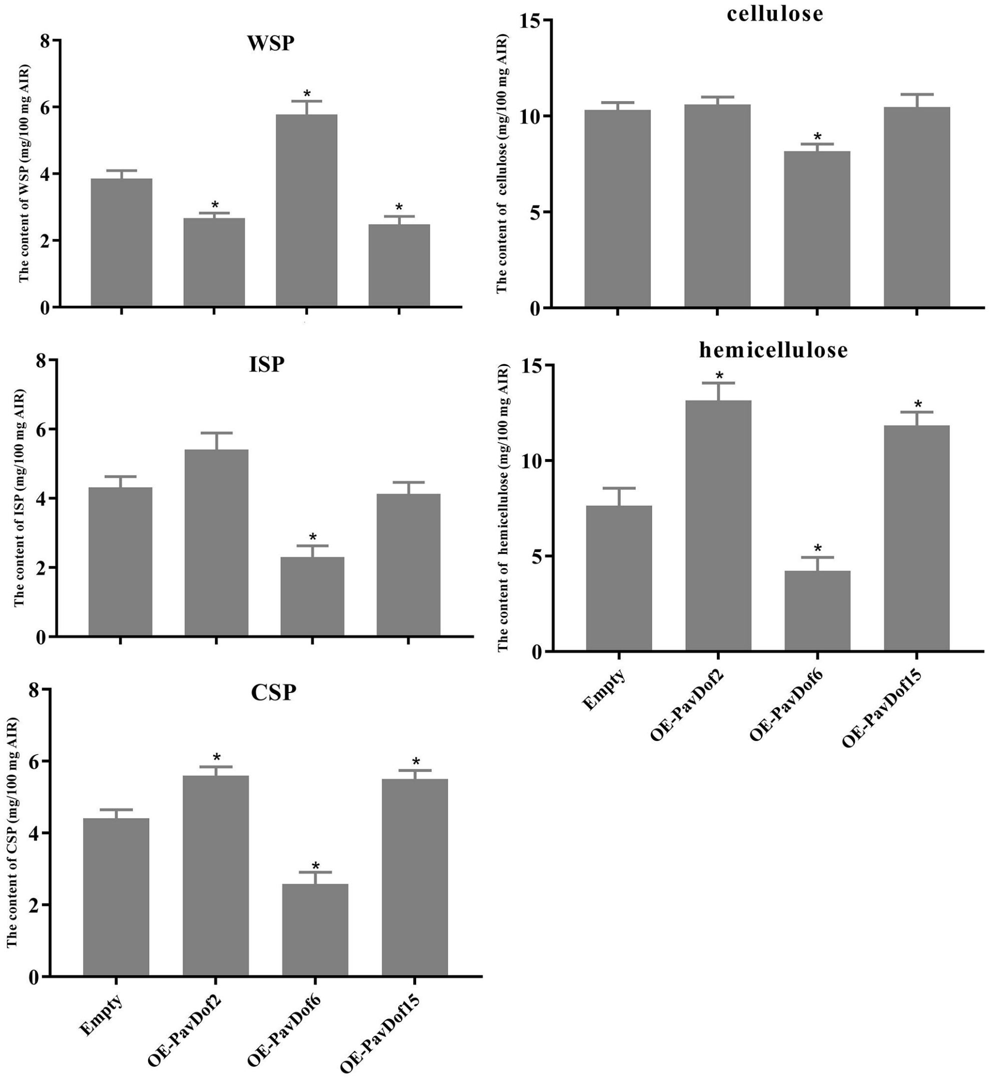

Supplemental Figure S10. Composition of the cell wall in transgenic cherry fruits. CSP, covalent pectin; WSP, water-soluble pectin; ISP, ion-linked pectin; AIR, alcohol-insoluble residues. The empty vector was used as a control. Data are shown as means ± SD of three biological replicates. Significant differences were determined by Student's t-test (***P* < 0.01, **P* < 0.05).

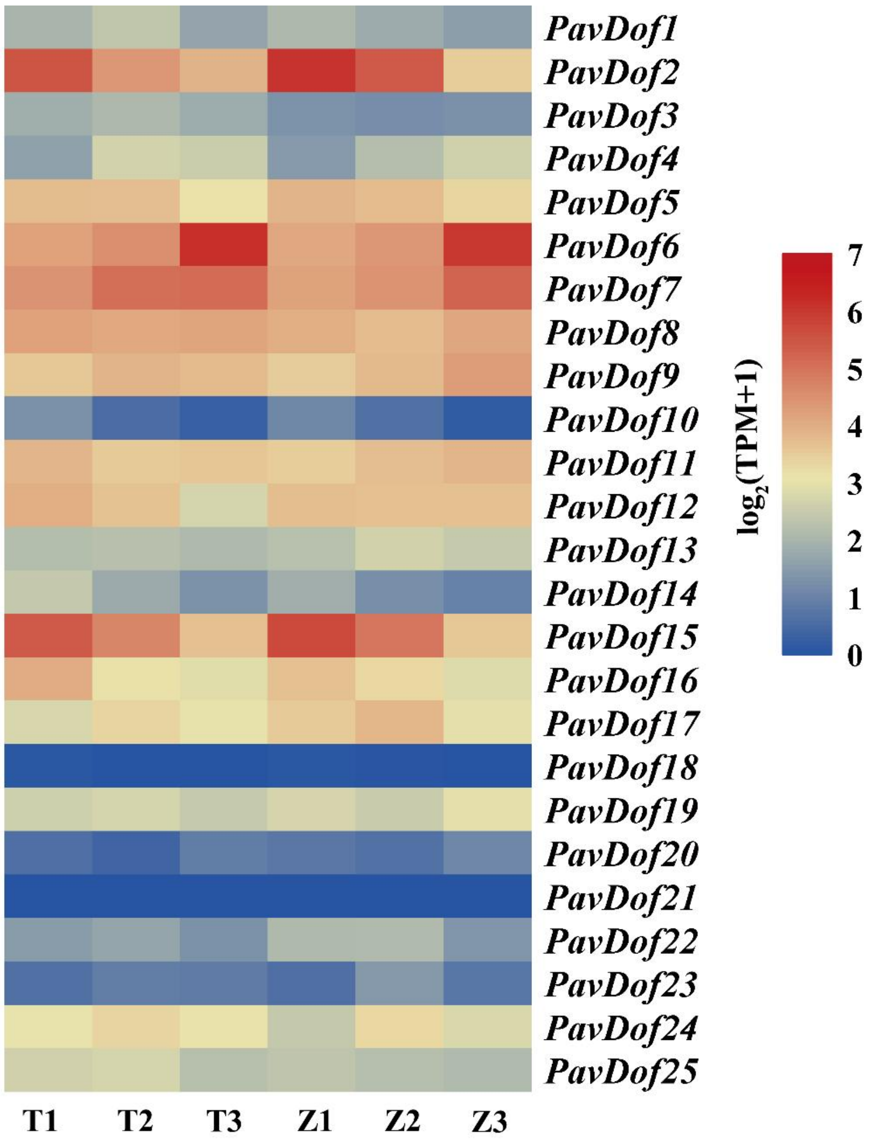

Supplemental Figure S11. Expression profile of *Dof* genes in sweet cherry. The colour scale represents expression levels as Log_2(TPM+1) across different samples. T, Tieton; Z, Zaodaguo; 1, big green (BG); 2, yellow white (YW); 3, full red (FR).

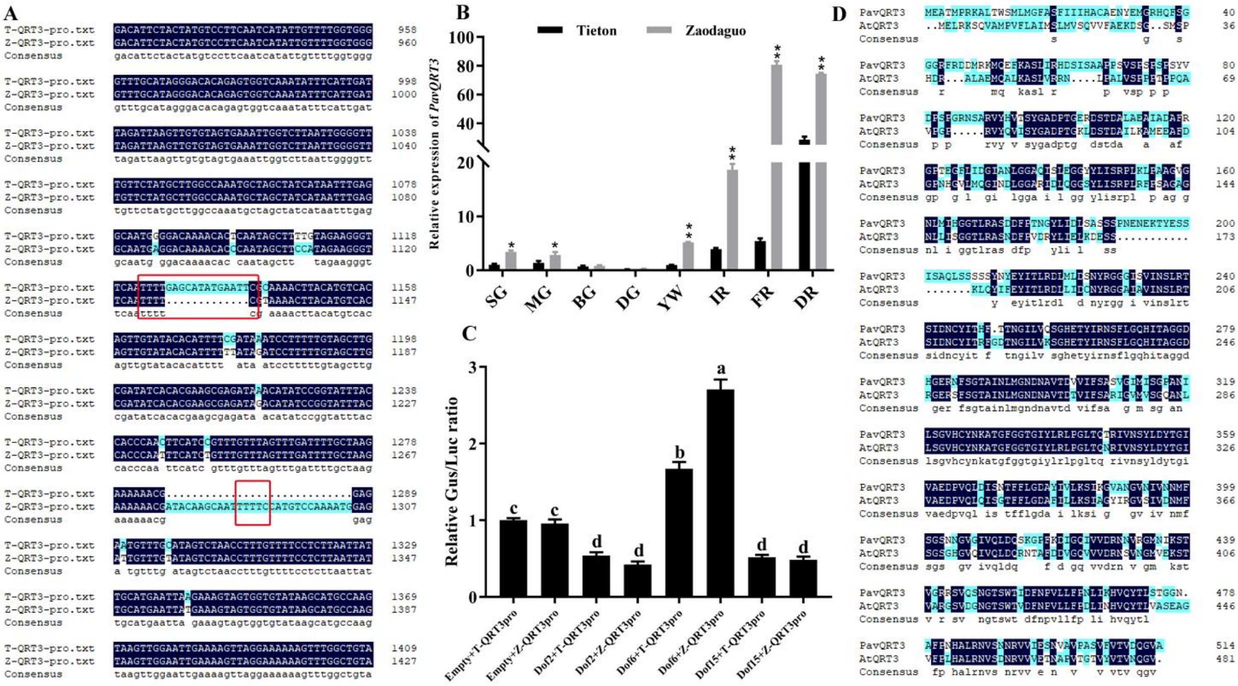

Supplemental Figure S12. Regulation of the *PavQRT3* promoter by PavDof2/6/15 from Tieton and Zaodaguo. A, Sequence alignment of the *PavQRT3* promoter sequence from Tieton and Zaodaguo. The red rectangle indicates two missing Dof binding sites in Tieton. **B,** Relative *PavQRT3* transcript levels in Tieton and Zaodaguo during fruit development. SG, big green; MG, mid green; BG, big green; DG, degreening; YW, yellow white; IR, initial red; FR, full red; DR, dark red. Data are shown as means ±SD from three replicates. Significant differences were determined by Student's t-test (**$P < 0.01$, *$P < 0.05$). **C,** Transient expression in *N. benthamiana* validating that PavDof2/6/15 regulate the *PavQRT3* promoters from Tieton and Zaodaguo. Data are shown as means ± SD from three replicates. Different letters refer to significant differences by Duncan's multiple range test with $P < 0.05$. **D,** Sequence alignment of PavQRT3 and Arabidopsis QRT3. For A and D, the navy blue represents consistent sequence and the light blue represents inconsistent sequence.

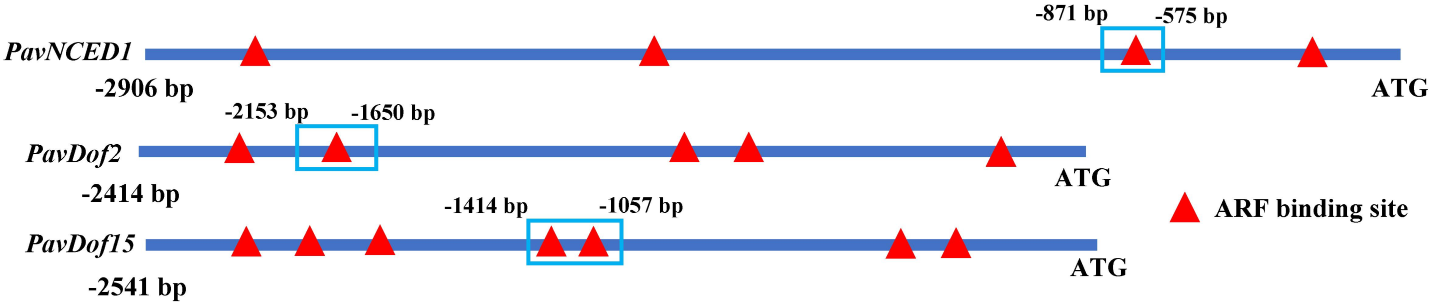

Supplemental Figure S13. Predictions of AuxREs in the promoters of *PavDof2/15* and *PavNCED1.* The blue lines refer to the promoter sequences of *PavDof2/15* and *PavNCED1*. The red triangles refer to ARF binding sites. The blue rectangles represent the promoter fragments used for Y1H.

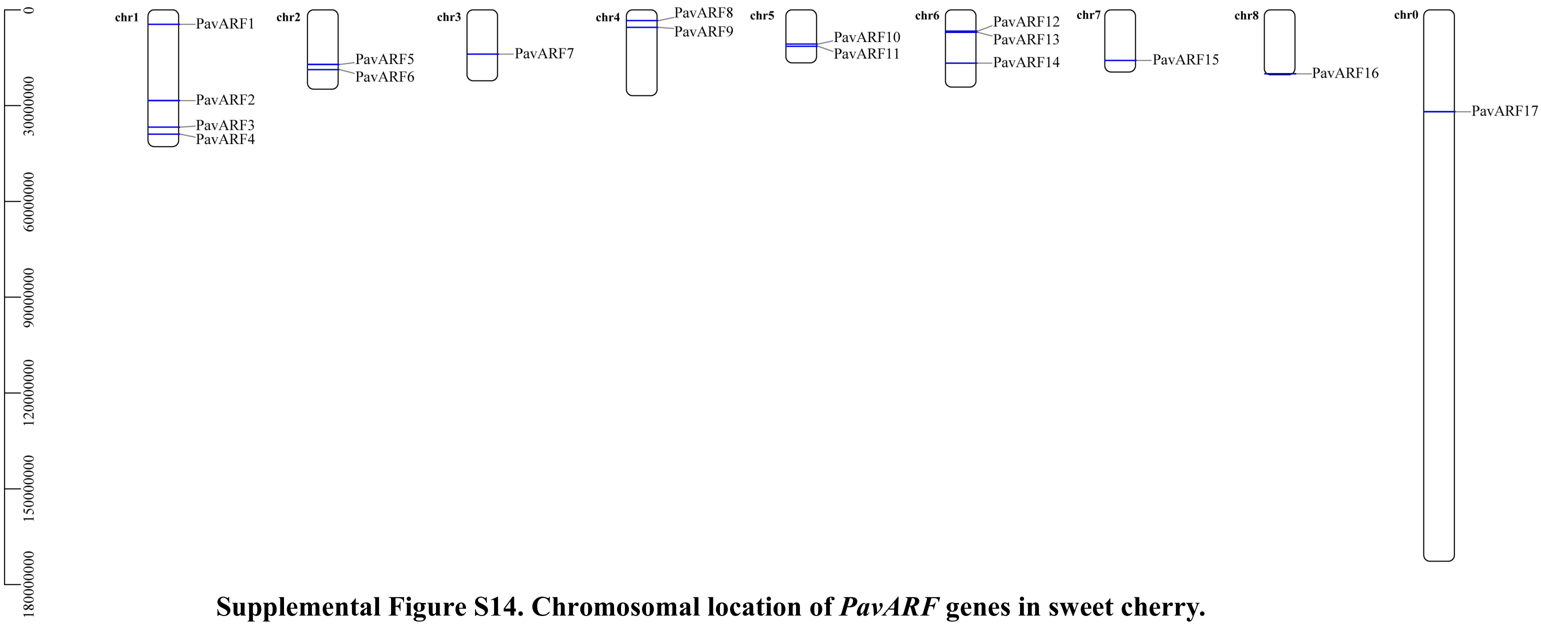

Supplemental Figure S14. Chromosomal location of *PavARF* genes in sweet cherry.

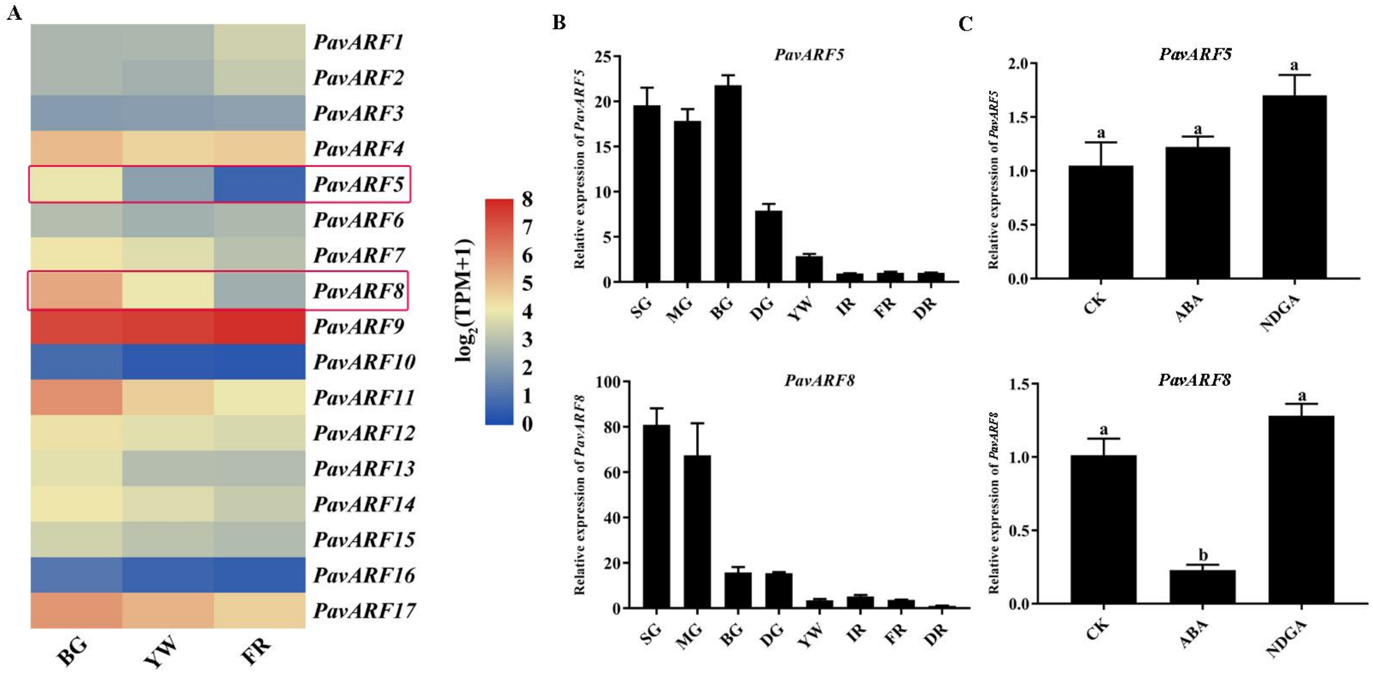

Supplemental Figure S15. The expression pattern of *PavARFs* in Zaodaguo. A, Heatmap representation of *PavARF* expression levels in Zaodaguo. The red rectangles indicate the candidate *PavARFs*. **B,** Relative *PavARF5* and *PavARF8* transcript levels during fruit ripening in Zaodaguo. Data are shown as means ± SD of three biological replicates, each biological replicate contains 15 fruits. SG, big green; MG, mid green; BG, big green; DG, degreening; YW, yellow white; IR, initial red; FR, full red; DR, dark red. **C,** Effect of ABA and NDGA treatment on the expression of *PavARF5* and *PavARF8*. *PavActin* (Genbank: FJ560908) was used as an internal control. CK, control check; ABA, abscisic acid; NDGA, nordihydroguaiaretic acid. Data are shown as means ± SD from three biological replicates. Different letters refer to significant differences by Duncan's multiple range test with $P < 0.05$.

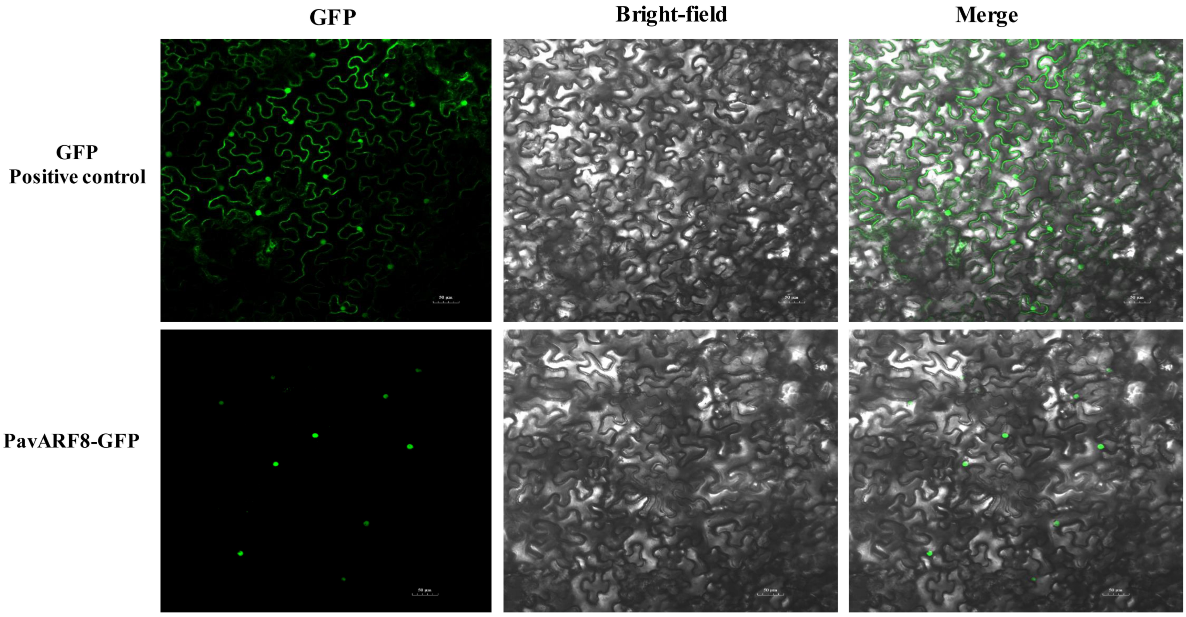

Supplemental Figure S16. Subcellular localization assay of PavARF8. *GFP* (Positive control) and the *PavARF8-GFP* construct driven by the CaMV 35S promoter were separately transiently infiltrated in epidermal cells of *N. benthamiana* leaves. Scale bars = 50 μm.
